# The RNA-binding protein TTP is a global post-transcriptional regulator of feedback control in inflammation

**DOI:** 10.1093/nar/gkw474

**Published:** 2016-05-24

**Authors:** Christopher Tiedje, Manuel D. Diaz-Muñoz, Philipp Trulley, Helena Ahlfors, Kathrin Laaß, Perry J. Blackshear, Martin Turner, Matthias Gaestel

**Affiliations:** 1 Institute of Physiological Chemistry, Medical School Hannover (MHH), 30625 Hannover, Germany; 2 Lymphocyte Signalling and Development, The Babraham Institute, Cambridge CB22 3AT, UK; 3 Laboratory of Signal Transduction, National Institute of Environmental Health Sciences, Research Triangle Park, NC 27709, USA; and Departments of Medicine and Biochemistry, Duke University Medical Center, Durham, NC 27710, USA

## Abstract

RNA-binding proteins (RBPs) facilitate post-transcriptional control of eukaryotic gene expression at multiple levels. The RBP tristetraprolin (TTP/Zfp36) is a signal-induced phosphorylated anti-inflammatory protein guiding unstable mRNAs of pro-inflammatory proteins for degradation and preventing translation. Using iCLIP, we have identified numerous mRNA targets bound by wild-type TTP and by a non-MK2-phosphorylatable TTP mutant (TTP-AA) in 1 h LPS-stimulated macrophages and correlated their interaction with TTP to changes at the level of mRNA abundance and translation in a transcriptome-wide manner. The close similarity of the transcriptomes of TTP-deficient and TTP-expressing macrophages upon short LPS stimulation suggested an effective inactivation of TTP by MK2, whereas retained RNA-binding capacity of TTP-AA to 3′UTRs caused profound changes in the transcriptome and translatome, altered NF-κB-activation and induced cell death. Increased TTP binding to the 3′UTR of feedback inhibitor mRNAs, such as Ier3, Dusp1 or Tnfaip3, in the absence of MK2-dependent TTP neutralization resulted in a strong reduction of their protein synthesis contributing to the deregulation of the NF-κB-signaling pathway. Taken together, our study uncovers a role of TTP as a suppressor of feedback inhibitors of inflammation and highlights the importance of fine-tuned TTP activity-regulation by MK2 in order to control the pro-inflammatory response.

## INTRODUCTION

RNA-binding proteins (RBPs) constitute an important group of post-transcriptional regulators in diverse cellular processes acting on both coding and non-coding RNAs (1,2). A prominent role for the post-transcriptional control by RBPs is emerging especially in the regulation of innate immunity (3). In this context the RBP tristetraprolin (TTP), encoded by the Zfp36 gene of the Tis11-family, acts on pro-inflammatory cytokine mRNAs. It guides them for degradation and/or prevents their efficient translation (4). This function depends on the RNA helicase Ddx6 (5) or the 4EHP-GYF complex (6). Distinct sequences such as AU-rich elements (AREs) in the 3′UTRs of mRNAs regulate their stability by serving as binding platforms for TTP (7). During inflammatory conditions triggered by TLR4-mediated activation of macrophages with lipopolysaccharide (LPS), these mRNAs are stabilized and their translation is preferentially enhanced (8). The molecular switch for this regulation relies on the phosphorylation of TTP by p38^MAPK^-activated protein kinase 2 (MAPKAPK2 or MK2) at two conserved serine residues (S52 and S178 in mouse, S60 and S186 in human) (9). This phosphorylation results in a sequestration of TTP by 14-3-3 proteins preventing its mRNA degrading activity (10) and might change TTP's RNA affinity to AREs (8). A phosphorylation-sensitive interaction of TTP with components of the Ccr4-Not1-deadenylation complex (11–13) and enhanced replacement of phospho-TTP by translation-promoting factors (8) was additionally shown. To better understand the global regulatory potential of TTP and the consequences of phosphorylation-dependent binding to its mRNA targets, we have applied a combined high-throughput sequencing approach.

TTP belongs to a group of negative feedback inhibitors that is crucial for the shutdown of the pro-inflammatory response, thereby preventing chronic inflammation (14). The essential role of these feedback inhibitors, such as TTP, dual specificity phosphatase 1 (Dusp1), immediate early response 3 (Ier3) and tumor necrosis factor alpha-induced protein 3 (Tnfaip3/A20), has been demonstrated by genetic deletions, which led to deregulated inflammatory responses and macrophage death (14–21). Similar to cytokines and chemokines, the expression of these feedback inhibitors is increased in macrophages in response to LPS due to the existence of AREs within their own 3′UTRs (14). Interestingly, the transcription of these feedback inhibitors often depends on NF-κB activity that itself is regarded as part of an auto-regulatory feedback loop (22,23). In turn, TTP also blocks nuclear import of NF-κB (p65) due to a direct physical interaction between the proteins and/or due to acting as a co-repressor on NF-κB (24,25).

TTP-deficient mice develop a complex inflammatory phenotype in part due to the overproduction of tumor necrosis factor (TNF) (20). By contrast, analysis of the *Zfp36^aa/aa^* transgenic mouse, in which the endogenous TTP gene was replaced by a non-MK2-phosphorylatable mutant TTP-S52, 178A (TTP-AA), showed attenuated systemic inflammatory response to LPS due to a decrease in cytokine production (26). Although in this *in vivo* approach the mutant TTP-AA is expressed to a much lower level than wild-type TTP, results indicate that TTP is a key regulator of inflammation. However, the global molecular pathways controlled by TTP are only partly understood due to the lack of comprehensive knowledge about TTP-bound mRNA targets. Previous studies, which combined RNA-immunoprecipitation with microarray analysis to identify TTP mRNA targets in mouse embryonic fibroblasts (MEFs) (27), macrophages (28), dendritic cells (29) or primary macrophages (30), have listed and validated TTP-dependent regulation of mRNA levels and translation for some TTP mRNA targets, but none of these studies provided information about direct physical TTP-nucleic acid interactions. Importantly, not all of the TTP targets identified contained AREs raising questions about the specificity of binding (27,29). Recently, photoactivatable ribonucleoside-enhanced-crosslinking immunoprecipitation (PAR-CLIP) was used to characterize TTP-RNA interactions on a transcriptome-wide scale in HEK293 cells (31). However, in this study the forced ectopic expression of TTP does probably not reflect the TTP-mediated post-transcriptional mRNA regulation in its physiological context, such as in LPS-stimulated macrophages (31–33).

Here, we report a genome-wide screen to determine the TTP-RNA interactome in bone marrow derived macrophages (BMDMs) using individual nucleotide resolution crosslinking and immunoprecipitation (iCLIP) (34) and high-throughput sequencing-based transcriptomic approaches. We have generated a protein expression rescue system in TTP-deficient macrophages that mimics endogenous protein expression of TTP after LPS stimulation. The system allowed us to address the effects of post-translational regulation of TTP by MK2 using an integrated systems biology approach by correlating changes of TTP binding to its mRNA targets with changes in mRNA abundance and translation during the early stages of macrophage activation. Our study has identified thousands of novel TTP targets, including mRNAs of cytokines and chemokines like Gdf15 and Cxcl10, and has also elucidated the global phosphorylation-dependent properties of TTP. Furthermore, our approach has uncovered the novel function of TTP as a suppressor of feedback inhibitors, such as Dusp1, Ier3 and Tnfaip3 that control the pro-inflammatory response triggered by LPS. Our observations underline the importance of MK2-dependent post-translational TTP regulation for correct NF-κB-signaling and cell survival.

## MATERIALS AND METHODS

### Cell culture

Generally, macrophages were grown in DMEM containing 2 mM l-glutamine, 10% (v/v) FCS, 100 U penicillin G/ml, 100 mg/ml streptomycin and non-essential amino acids (all purchased from Life Technologies) in a humidified atmosphere at 37°C. To obtain bone marrow-derived macrophages, femurs of 6 week-old mice were flushed and plated on one 10 cm plate. 10 ng/ml M-CSF (Wyeth/Pfizer) was added to the medium. The next day non-adherent cells were transferred to a new 10 cm plate and new medium containing 10 ng/ml M-CSF was added to the initial plate. Medium was renewed on both plates after another 3 and 6 days. Cells were scraped and seeded for experiments on day 9 to 10 after initial plating. Primary TTP-deficient (20) macrophages were immortalized, stained for macrophage-specific surface markers and subsequently sorted by FACS as described previously (35). Immortalized bone marrow derived macrophages (BMDMs) were transduced with retroviruses containing an all-in-one tet-inducible vector (36) coding for GFP–TTP, GFP–TTP-AA or GFP-only constructs (for cloning primers see Supplementary Table S4). Of note, only protein coding sequences for TTP were inserted in this constructs to avoid autoregulation of TTP through binding to its own mRNA 3′UTR (37). Plasmids coding for TTP and TTP-AA described before (8) served as templates for amplification. Transductions were carried out as described before (35). Doxycycline and LPS (Sigma, *E. coli* O127:B8) were used at 1 μg/ml for cell stimulation. The TTP-deficient MEF cell line was described before (27). HEK293T, HeLa and RAW264.7 cells were maintained and transfected as previously described (38).

### Animals

Wild-type (C57BL/6) and Zfp36^tm1Pjb^ (TTP^−/−^) mice (20) were bred from heterozygotes and experiments were conducted according to German and international guidelines and were approved by the ethics committee of Hannover Medical School (MHH). Genotyping of mice was done using the wild-type specific primers TTPd and TTPrc1 and knockout-specific primers TTPd and Tneorc (for primer sequences see Supplementary Table S4).

### Flow-cytometry

Cells were scraped in medium and briefly vortexed before the analysis. An Accuri (Becton Dickinson) system was used to check the immortalized cells for macrophage-specific F4/80-PE (eBiosciences, clone BM8) and CD11b-PE (eBiosciences, clone M1/70) surface marker expression. To control successful transduction, immortalized cells were analyzed for GFP-expression upon doxycycline-treatment. Sorting of GFP-positive cells upon 6 h of doxycycline-treatment was carried out by the FACS Sorter Core Facility at the Hannover Medical School.

To measure apoptotic cells, 10 μg/ml 7-amino actinomycin D (7-AAD, Life Technologies) (39) was added to 0.2-0.5 × 10^6^ cells collected in 100 μl FACS-buffer (1 × PBS, 2 mM EDTA) and incubated for 15 min at 4°C before determining the percentages of 7-AAD^+^ cells. Here, after gating on viable cells in the SSC-A/FSC-A scatter-plot, GFP^+^ cells were gated and checked for the percentage of 7-AAD^+^ GFP^+^ cells. The mean and the corresponding standard deviations of three independent experiments are shown.

### Fluorescence microscopy

Cells were grown on poly-L-lysine coated (50 μg/ml) coverslips for 24 h. After stimulation with doxycycline for 6 h, cells were treated with LPS for the indicated times and immediately fixed with 4% paraformaldehyde (PFA, in 1× PBS) for 15 min at room temperature. After blocking with 5% BSA in 1× PBS containing 0.3% Triton X-100, staining was done with a 1:400 dilution of the anti-p65 (NF-κB) antibody (D14E12) XP (Cell Signaling Technology) for 2 h at room temperature. An anti-rabbit secondary antibody coupled to Alexa Fluor-555 (Molecular Probes, 1:400 dilution in 1× PBS containing 0.3% Triton X-100) was used to detect the anti-p65 antibody (2 h at room temperature) together with Alexa-Fluor 647 Phalloidin (Molecular Probes, 1:400 dilution) to stain for F-actin before nuclear staining with a DAPI-solution (500 ng/ml) for 10 min at room temperature. Images were taken on a confocal Leica TCS SP2 microscope at the central imaging facility of Hannover Medical School (MHH).

Quantitative image analysis of NF-κB nuclear import upon LPS stimulation was done using the ImageJ software (http://rsb.info.nih.gov/ij/index.html) with the help of the plug-in JACoP. Mander's coefficients were determined for cytosolic (F-actin co-staining) and nuclear (DAPI co-staining) NF-κB localization in the cells. For each genotype and time point, five independent multi-channel images were taken and analyzed in a blind approach.

### Water soluble tetrazolium (WST-1) proliferation assay

The phosphate-buffered saline solution (Roche # 11 644 807 001) was added directly to the cultured cells and incubated for a minimum of 30 min at 37°C. 10 μl of the solution were used for 100 μl cell culture medium. Cells were seeded in triplicates in a 96-well format at a density of 1 × 10^4^ cells/well. Measurements at 450 and 630 nm for background subtraction were carried on a multiplate-reader (Wallac).

### SDS-PAGE, western blot and antibodies

SDS-PAGE and western blotting was carried out as described before (40). Western Blots were developed using the ECL-based LAS-3000 (FujiFilm) and the Odyssey scanner (Li-COR Biosciences) system, respectively. The anti-GAPDH was purchased from Millipore and the anti-Tubulin from Sigma. Anti-GFP, anti-β-Actin, anti-pro-TNF, anti-Tnfaip3, anti-Dusp1 and anti-Ier3 were all from Santa Cruz Biotechnology whereas all other antibodies were obtained from Cell Signaling. The anti-TTP (SAK21B) and anti-pTTP^S178^ antibodies were described before (41,42) and kindly provided. HRP-coupled secondary antibodies were obtained from Dianova and IRDye-680/800-coupled secondary antibodies were from Li-COR Biosciences.

For quantification of western blot signals a minimum of two different exposures was normalized to at least three different exposures of the loading control blot that was derived from the same membrane. All background-reduced band intensities were determined using the Multi Gauge V3.2 software (FujiFilm).

### Generation of rat-derived TTP-specific (NE2/1.1) and anti-pan-Tis11 antibodies

Rats were immunized with the N-terminal 84 amino acids of TTP starting with SLQSMS and fused to GST. The resulting antibody (NE2/1.1, IgG_1_) was used in a 1:1000 dilution for the detection of TTP in western blot. To generate the anti-pan-Tis11 antibody, rats were immunized with the C-terminal 164 amino acids of Zfp36l1 (or Tis11b) fused to GST. The resulting IgG_2a_ was used 1:1000 in western blot and detected TTP, Zfp36l1 and Zfp36l2.

### ELISA

For the detection of mouse Cxcl10 chemokine the DuoSet kit #DY466-05 (R+D Systems) was used according to the manufacturer's instructions. 1 × 10^4^ cells were seeded in triplicates in a 96-well plate. After attachment to the bottom of the wells, cells were stimulated as indicated.

### Luciferase reporter assay measurement

3′UTR Firefly reporter plasmids (see Supplementary Table S4 for cloning primers) were co-transfected into HeLa cells together with a Renilla-coding plasmid and TTP or TTP-AA cloned into pEGFP-C1 (described in (43)) following previous descriptions (38).

### RNA-Isolation, cDNA synthesis and qRT-PCR

RNA was isolated using either the NucleoSpin RNA extraction kit (Macherey+Nagel) or TRIzol (Life Technologies) according to the manufacturer's instructions. cDNA from 250 to 500 ng RNA was synthesized using the first strand cDNA synthesis kit (Fermentas/Thermo) in combination with random hexamer primers. qRT-PCRs were run on a Rotor-Gene-Q (Qiagen) device using either 2× Probe or 2× SYBR-Green SensiFast mixes (no ROX, Bioline) according to the instructions. Primers used in this study are listed in Supplementary Table S4.

### RNA-stability measurements

10 μg/ml Actinomycin D (Sigma) were added after stimulation of cells to stop the *de novo* transcription by RNA Polymerase II. Samples for RNA-isolation were taken every 15 min after the initial addition of Actinomycin D. RNAs were further processed as described before (40).

### RNA-immunoprecipitation (RNA-IP)

1×10^6^ cells for each individual IP were seeded in 6 cm plates one day before stimulation with 1 μg/ml Doxycycline (6 h) and LPS (1 h). anti-GFP IPs were performed in the presence of 0.5 mg/ml heparin (Carl Roth), Phosphatase Inhibitor Cocktail III (Sigma) and protease inhibitors (Roche) according to the instructions described before (44) and monitored by GFP western blot (Supplementary Figure S4a). Precipitated RNAs were eluted using TRIzol and equal volumes of eluted RNA were purified and converted into cDNA. Relative levels of precipitated RNA in relation to the control GFP-IP were determined by qPCR using the primers listed in Supplementary Table S4.

### Polysome profiling

Polysome profiling of individual mRNAs was described before (8), with the exception that total lysates were used. Macrophages were rinsed in ice cold 1× PBS containing 0.1 mg/ml cycloheximide prior to lysis. Total lysates from 10 × 10^6^ cells were separated on a linear 10–50% sucrose gradient by ultracentrifugation for 2 h at 35 000 rpm using a SW40.1 Ti rotor (Beckman-Coulter). After centrifugation 12 fractions (1 ml each) per gradient were collected using a UA-6 UV/VIS (Teledyne/ISCO Inc.) device. The RNA of each fraction was precipitated overnight with isopropanol and 3 M Na-acetate at –20°C and purified using the NucleoSpin RNA kit (Macherey+Nagel) by dissolving the resulting pellet in buffer RA1 from the kit. Purified RNAs were subjected to cDNA synthesis and qRT-PCR as described above.

### Ribosome profiling (RiboSeq) and total RNA sequencing (RNASeq)

Genome-wide assessment of ribosome-occupancy was assessed using ARTSeq Ribosome profiling kit (Epicentre). Briefly, 10 × 10^6^ macrophages were seeded on a 15 cm plate the day before induction of TTP expression with doxycyclin (6 h treatment) and stimulation with LPS for 1 h. Cells were rinsed twice with ice cold 1× PBS containing cycloheximide (0.1 mg/ml) before cell extract preparation (≈1 ml). One tenth (100 μl) of the sample was used for total RNA isolation and library preparation using the Ribo-Zero Gold Kit (Epicentre) and following instructions in the ARTSeq Ribosome profiling kit. 200 μl of the samples were used for nuclease digestion and ribosome-protected RNA purification prior to cDNA library preparation. cDNA library size was assessed using High Sensitivity DNA Analysis BioAnalyzer kits (Agilent) and libraries were quantified using KAPA Library Quantification Kits (Kapa Biosystems). 50 bp SE multiplexed sequencing was done on an Illumina HiSeq2500 device.

### Individual-nucleotide resolution crosslinking and immunoprecipitation (iCLIP)

iCLIP experiments were performed as previously described (34). For each experiment 10 × 10^6^ cells were seeded a day before treatment with doxycycline (1 μg/ml) for 6 h accompanied by 1 hour stimulation with LPS (1 μg/ml). After washing with ice-cold PBS, intact cells were irradiated with UV-C light (300 mJ/cm^2^, Stratalinker 2400) and total cell extracts were obtained using lysis buffer (50 mM Tris/HCl [pH 8.0], 150 mM NaCl, 0.5% (v/v) Triton X-100 and 1 mM EDTA) and sonication (3 times for 20 s in ice). Extract were cleared by centrifugation (15 min at 4°C) and treated with RNase A (0.9375 × 10^−3^ Units/ml, Roche) and DNase I (20 Units/ml, Ambion) for 3 min at 37°C and cooled on ice for 5 min after adding urea to 0.5 M and RNase inhibitors (266.8 Units of RiboLock, Thermo/Fermentas; and 133.4 U of RNaseOUT, Life Technologies). Immunoprecipitation of GFP–TTP-RNA complexes was performed using 25 μl of Epoxy Dynabeads (M-270, Life Technologies) coupled GFP-nanobody slurry per 1 ml of total cell extracts for 1 h at 4°C. Coupling was described before (45). Samples were washed with lysis buffer plus 0.5 M Urea (1 x) and low salt-urea wash buffer (50 mM Tris/HCl [pH 8.0], 150 mM NaCl, 0.1% (v/v) Triton X-100 and 0.5 M Urea) (3x). Samples were vortexed and kept in rotation for 5 min between washes. Next, samples were further washed using high salt-urea wash buffer (50 mM Tris/HCl [pH 8.0], 1000 mM NaCl, 0.1% (v/v) Triton X-100 and 4 M Urea) (2×) and PNK wash buffer (20 mM Tris–HCl [pH 7.4], 10 mM MgCl_2_, 0.2% Tween-20) (3×). Samples were changed to a new tube before RNA dephosphorylation by suspending the slurry in RNA dephosphorylation mix (0.5 μl PNK [NEB], 1 μl RNase Inhibitor, 2 μl 10× PNK pH 6.5 buffer [NEB] and 17.5 μl water). One tenth of the sample was labeled with ^32^P-ATP using PNK, whereas an RNA Linker was ligated to 3′ end of the RNA molecules present in the rest of the sample. Then, GFP–TTP-RNA complexes were resolved by electrophoresis (SDS-PAGE) and transferred to nitrocellulose membranes. Visualization by autoradiography of radioactively labeled RNA was performed prior to isolation of GFP–TTP-RNA complexes larger than 75 kDa by incubating the nitrocellulose fragment at 37°C for 10 min with proteinase K (0.14 mg, Roche) in 200 μl of PK buffer (100 mM Tris/HCl [pH 7.5], 50 mM NaCl and 10 mM EDTA). Additional 200 μl of PK buffer plus 7 M urea was added and samples were further incubated at 37°C for 20 min. RNA isolation was performed by phenol/chlorophorm extraction and ethanol precipitation, prior retro-transcription of the RNA into cDNA by using SuperScript III reverse transcriptase and RCLIP primers with a seven bases barcode (3 know + 4 unknown). cDNA was gel-purified (6% TBE-urea gels), circularized using Circligase II and amplified by PCR using Solexa P5/P7 primers. cDNA libraries from independent experiments were prepared and sequenced using a HTSeq2500 (50 bp SE sequencing).

### Bioinformatics

iCLIP data analysis was performed as previously described (34). Briefly, sample demultiplexing was performed by identification of the three known bases of the seven bases barcode introduced in the 5′ end of the read by the RCLIP primer. The remaining four unknown bases were used for identification of unique cDNA counts after removal of PCR duplicate reads. Reads were trimmed to remove any adaptor sequence and barcodes before mapping unique reads to genome mm10 (ensembl75 annotation) using Bowtie. Position -1 was extracted and annotated as the TTP crosslink site and associated to the different genomic regions (ncRNA, protein-coding genes, telomers, intergenic) as defined by Ensembl. Crosslinks within regions of protein-coding genes were further assigned to 5′UTR, ORF and 3′UTR. Peak enrichment analysis of TTP binding sites was performed in iCount with the sum of unique TTP crosslink sites identified in the different number of independent experiments as previously described (34). Briefly, 15 nucleotide windows flanking the crosslink site were generated, 100 permutations were allowed and multiple test correction of *P*-values (Benjamini-Hochberg) was performed to calculate false-discovery rates (FDR). Only TTP crosslink sites with a FDR lower than 0.05 were further considered in our analysis. Bed files summarizing iCLIP mapped data for the groups of GFP–TTP (1) and GFP–TTP-AA (2) upon 6 h Dox/1h LPS after peak enrichment (FDR < 0.05) are provided as Supplementary Files 1 and 2.

For k-mer analyses, crosslinks associated to ncRNA or 3′UTR were considered separately. Prior to analysis, reads in the antisense transcriptional direction were removed and 20 nucleotides windows were generated in both directions 10 nt far from each crosslink to finally assess frequency of k-mer occurrency in a 100 nt-long windows (-50, +50). 100 randomized iCLIP positions present in the same genomic feature were used to calculate the random mean score. *Z*-score was calculated as the score frequency of a given 5-mer occurrence normalized by the random mean score.

RNA maps were calculated after definition of ORF-3′UTR and 3′UTR-intergenomic junctions in genome mm10 (ensembl75 annotation) and after the generation of windows with the 300 nt upstream and downstream each junction. The position of TTP crosslink sites in each window is shown after normalization by the total number of crosslinks and by all used junction spanning positions (to correct for differences in length).

Raw data from RNASeq and RiboSeq was demultiplexed, FastQ analyzed and mapped to mm10 genome (Mus_musculus.GRCm38. 70 .gtf annotation) with TopHat (v2.0.7) using tophat -p 6 -g 2 parameters. Differential gene expression analysis was performed using DESeq2_1.6.3 package (46) and was adjusted to a negative binomial distribution after correction by library size factor before testing for differential expression analysis, *P*-value determination and correction by using a Benjamini-Hochberg multiple test to obtain final *P*-adjusted (padj) values. A multi-factor design was used to account for the three different conditions and for putative experimental batch effects [DeSeq2 parameters: design(dds) ← formula(∼ Library_date + Condition); nbinomWaldTest and Cooks filter off]. iCLIP, RNASeq and RiboSeq data were either visualized in UCSC genome viewer or in the Integrated Genome Browser (IGV) to show read coverage and search for AU-rich elements within 3′UTRs. Gene ontology (GO) analyses were performed using GOrilla (47) whereas pathway enrichment analysis was performed using WebGestalt in which a hypergeometric statistical test followed by Benjamini-Hochberg correction of the *P*-values was performed. Only pathways with more than five genes were considered (48). Alternatively, the gene set enrichment analysis tool (49) was used to analyze gene sets of specific enriched terms.

### Statistics for non-sequencing data

Values in bar graphs are shown as the mean of a minimum of three measurements with their standard deviations. An unpaired *t*-test was used where indicated. *P*-values <0.05 were considered significant.

## RESULTS

### Inducible expression of TTP and the phosphorylation site mutant TTP-AA in TTP-deficient mouse macrophages

**Figure 1.**
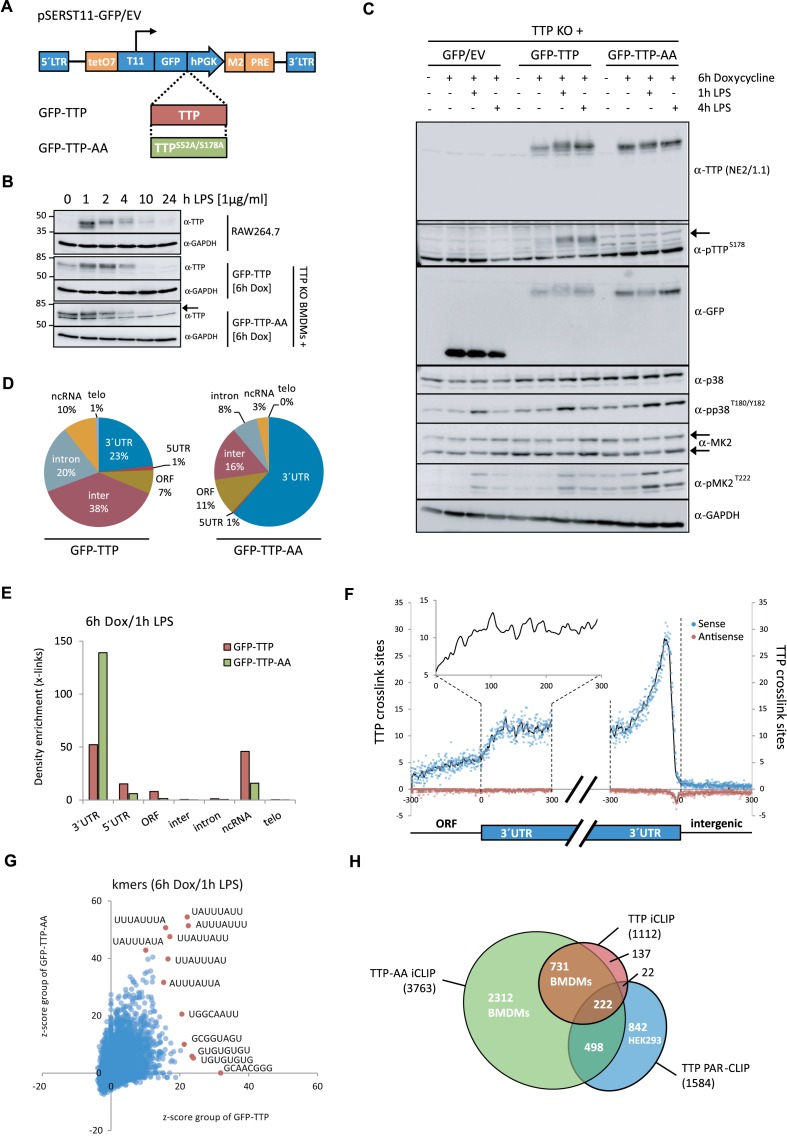
Expression of TTP and TTP-AA in macrophages and TTP-iCLIP. (**A**) Schematic representation of the all-in-one inducible vector pSERST11 that allows the expression of GFP, GFP–TTP or GFP–TTP-AA upon addition of doxycycline. (**B**) Comparison of the TTP induction kinetics in RAW264.7 cells with GFP–TTP- and GFP–TTP-AA expressing mouse macrophages after LPS stimulation for the indicated times. GFP–TTP and GFP–TTP-AA expression was induced by pretreating the cells with doxycycline for 6 h prior to LPS addition. The arrow indicates the position of GFP–TTP-AA protein while the lower band in the same bot is unspecific. (**C**) FACS-sorted GFP-positive cells were treated with either doxycycline alone for 6 h or in combination with LPS for 1 and 4 h, were lysed and analyzed by western blot for the indicated proteins. (**D**) Transcriptome-wide distribution of crosslinks in RNAs from GFP–TTP and GFP–TTP-AA iCLIP experiments upon 6 h doxycycline induction in combination with 1 h LPS. Results from individual experiments were grouped (GFP–TTP *n* = 4 and GFP–TTP-AA *n* = 3). The fraction of unique TTP crosslink sites mapped to the 3′ or 5′ untranslated region (UTR), open reading frame (ORF), intergenic regions, introns or telomeric regions is presented as the percentage of the sum of unique cDNA counts (for peak call analysis (false-discovery rate, <0.05)). (**E**) Density enrichment (by normalization of the data from (D) to the total length of the genomic features) for the GFP–TTP- and GFP–TTP-AA-bound RNAs to the different transcriptome subsets after the indicated stimulation (GFP–TTP *n* = 4 and GFP–TTP-AA *n* = 3). (**F**) The number of crosslinks in the sense (blue dots) and antisense direction (red dots) for the relative distance (-300 to 300) to the ORF-3′UTR (left) and 3′UTR-intergenic junctions (right), respectively, are plotted and result in a smoothed distribution of crosslinks within this genomic feature (black line). The insert shows a higher resolution of the smoothed distribution in the upstream 3′UTR. (**G**) Determination of the preferred GFP–TTP and GFP–TTP-AA binding motifs upon 6 h doxycycline/1 h LPS stimulation by k-mer analysis. The k-mer-sequences of eight nucleotides in length with the highest abundance are labeled in red. All groups were derived from four and three individual iCLIP analyses, respectively. (**H**) Overlap between GFP–TTP and GFP–TTP-AA 3′UTR iCLIP targets and with TTP targets identified by PAR-CLIP (31). All targets with more than one cDNA count were considered. Venn diagrams were generated using the eulerAPE_v3 tool (84). The numbers of different targets in the groups are indicated.

For the iCLIP analysis of TTP in macrophages, we generated a cell system that mimics the induction of TTP expression after inflammatory activation. Immortalized TTP-deficient bone marrow-derived macrophages (BMDMs) (20) were transduced using retroviral constructs, which harbor a GFP-fusion of wild-type murine TTP or a non-MK2-phosphorylatable mutant of TTP (TTP-S52A, 178A or TTP-AA) downstream of a doxycycline-inducible promoter (Figure 1A). Both GFP–TTP and GFP–TTP-AA were expressed with similar kinetics (Figure 1B) and to a similar extent in these cells (Figure 1c) before and after LPS stimulation, respectively. We analyzed the kinetics of doxycycline/LPS-induced TTP and TTP-AA expression in the transduced cells in comparison with LPS-induced induction of TTP in RAW264.7 cells together with TTP's suppressive effects on TNF expression (Figure 1B and Supplementary Figure S1). Treatment conditions for the transduced macrophages (4–6 h doxycycline, 1 h LPS) resembled TTP expression and phosphorylation in RAW264.7 monocytes after ∼2 h indicating that we generated a functional rescue system with basal TTP expression in doxycycline-only treated cells (Figure 1B).

Next, we tested LPS-induced MK2-mediated phosphorylation of GFP–TTP and GFP–TTP-AA at S178, the only site of TTP for which phosphorylation-specific antibodies were available to us. Phosphorylation of GFP–TTP after stimulation with LPS was detected by the multiple band patterns and the shift to slower migrating bands (Figure 1C). Phosphorylation at S178 of GFP–TTP was detected after 1h and sustained throughout 4 h of LPS stimulation, but was not detected in macrophages expressing GFP–TTP-AA. Overall TTP phosphorylation (not only at S178), as detected by a gel-shift in the anti-GFP immunoblot (Figure 1C), showed a maximum for the wild-type protein after 1h stimulation with LPS and was still detectable, but to a lower extent after 4 h. The activity of the upstream kinases p38^MAPK^ and MK2 peaked after 1 h (Figure 1C). Notably, in GFP–TTP-AA expressing cells phosphorylation of p38^MAPK^ and MK2 was slightly increased and sustained giving a first indication of a role for TTP phosphorylation in the feedback regulation of the LPS response. However, here we cannot rule out that this effect could be also due to induction of TTP-AA already 6 h before LPS stimulation (Figure 1B).

### iCLIP of TTP and TTP-AA

We applied a high-throughput sequencing approach, iCLIP, to map the RNA-binding sites of both GFP–TTP and GFP–TTP-AA at single nucleotide resolution before and after macrophage stimulation with LPS. Effective immunoprecipitation (IP) of the protein of interest is essential to map RNA:protein interactions using iCLIP and since specific high-affinity TTP antibodies were not available, we used Llama-derived nanobodies, which efficiently bind and precipitate GFP-fusion proteins under both native and denaturing conditions (Supplementary Figure S2a) (44). After RNase A digestion (Supplementary Figure S2b) TTP–RNA complexes above 100 kDa were isolated and used for iCLIP library preparations (Supplementary Figure S2c).

### Genomic regions and RNA-binding motifs

First, we analyzed the distribution of TTP crosslink sites mapped to distinct genomic features after 1h of LPS stimulation. TTP bound to various genomic regions, such as 3′UTRs, ORFs, introns, intergenic regions and ncRNAs. Interestingly, the binding of TTP to 5′UTRs of mRNAs was rather rare. A remarkably increased association of TTP-AA with 3′UTRs compared to wild-type TTP was observed (Figure 1D). Quantitation of TTP crosslink density enrichment by normalization of the data to the total length of the genomic features confirmed the increased association of TTP-AA with 3′UTRs after 1 h LPS stimulation. It indicated that both GFP–TTP and GFP–TTP-AA bound to 3′UTRs (and ncRNAs) with higher frequency, compared to intronic and intergenic regions (Figure 1E). The increased binding of TTP-AA to the 3′UTRs is specific for 1 h LPS stimulation, whereas only minor differences between TTP and TTP-AA binding were observed in untreated cells or after treatment with LPS for 4 h (Supplementary Figure S3), a time when MK2 is inactive and overall phosphorylation of TTP is decreasing (cf. Figure 1C). Taken together, these findings indicate a specific and transient phosphorylation-dependent suppression of TTP binding to the 3′UTR of its mRNA targets and increased potential binding of phospho-TTP to other genomic regions/features, such as ncRNAs.

We further determined the relative position of the TTP crosslinks within the 3′UTRs. We found that the density of crosslinks mapped to 3′UTRs increased by two fold when compared to the density in ORFs (Figure 1F). The highest density of crosslinks in the 3′UTR was detected in the vicinity of the polyadenylation signals (AAUAAA) (Figure 1F), which are usually located ∼20–30 nucleotides (nt) upstream of the 3′ends of 3′UTRs (50).

Since iCLIP analysis is capable to determine the protein-binding site with single nucleotide resolution directly from the sequenced library, we determined the binding site sequence preferences of TTP and TTP-AA in the 3′UTR (Figure 1G). Interestingly, the frequency of TTP and TTP-AA binding to specific RNA sequences clearly differs as seen by the shape of the cloud and the indicated sequences of the k-mer analysis performed for octameric binding motifs (Figure 1G). After 1h of LPS treatment the most frequent TTP-RNA crosslinking sites for TTP-AA were found to correspond perfectly to the core of the ideal ARE UUAUUUAUU. In contrast, for wild-type TTP the frequency of binding to these ARE-sites was lower and additional binding to G-rich patterns, such as UGUGUGUGU or GCUUCGGG, was observed in the 3′UTRs, indicating that the phosphorylation of TTP may regulate its RNA-binding properties to AREs in the 3′UTR.

### Transcripts targeted by TTP and TTP-AA in macrophages and HEK cells

Preserved RNA-binding of the non-phosphorylatable mutant GFP–TTP-AA was reflected in the higher number of RNA targets identified for this protein compared to the number of wild-type GFP–TTP targets (Figure 1h). In total 3763 mRNAs with more than one crosslink in any genomic feature were identified for GFP–TTP-AA, whereas the corresponding number for wild-type TTP was only 1112 (Supplementary Data Set 1). RNA target comparison between wild-type TTP and mutant showed an 85% to 97% overlap depending on stringency, defined as the number of unique cDNA counts associated to a TTP cross link site (Supplementary Table S1). Comparison of our iCLIP datasets with a previously published PAR-CLIP study for FLAG/HA-tagged TTP in HEK293 cells (31), showed that 45% of the RNA targets identified by PAR-CLIP were also identified as targets in the TTP-AA iCLIP dataset and only 15% of these RNA targets were within our wild-type TTP iCLIP dataset (Figure 1h). This suggests that the PAR-CLIP study could have identified other-relevant mRNA targets specific for HEK293 cells. The high percentage of mRNA target overlap between GFP–TTP and GFP–TTP-AA indicated that both wild-type and phospho-mutant proteins bind a common set of targets, but possibly with different affinities, which are modulated through MK2-dependent TTP phosphorylation. Indeed, the large number of targets unique for GFP–TTP-AA in BMDMs treated with LPS for only one hour indicated a marked down-regulation of target binding by phosphorylation of the wild-type protein.

### Global consequences of TTP- and TTP-AA-binding for the myeloid transcriptome

**Figure 2.**
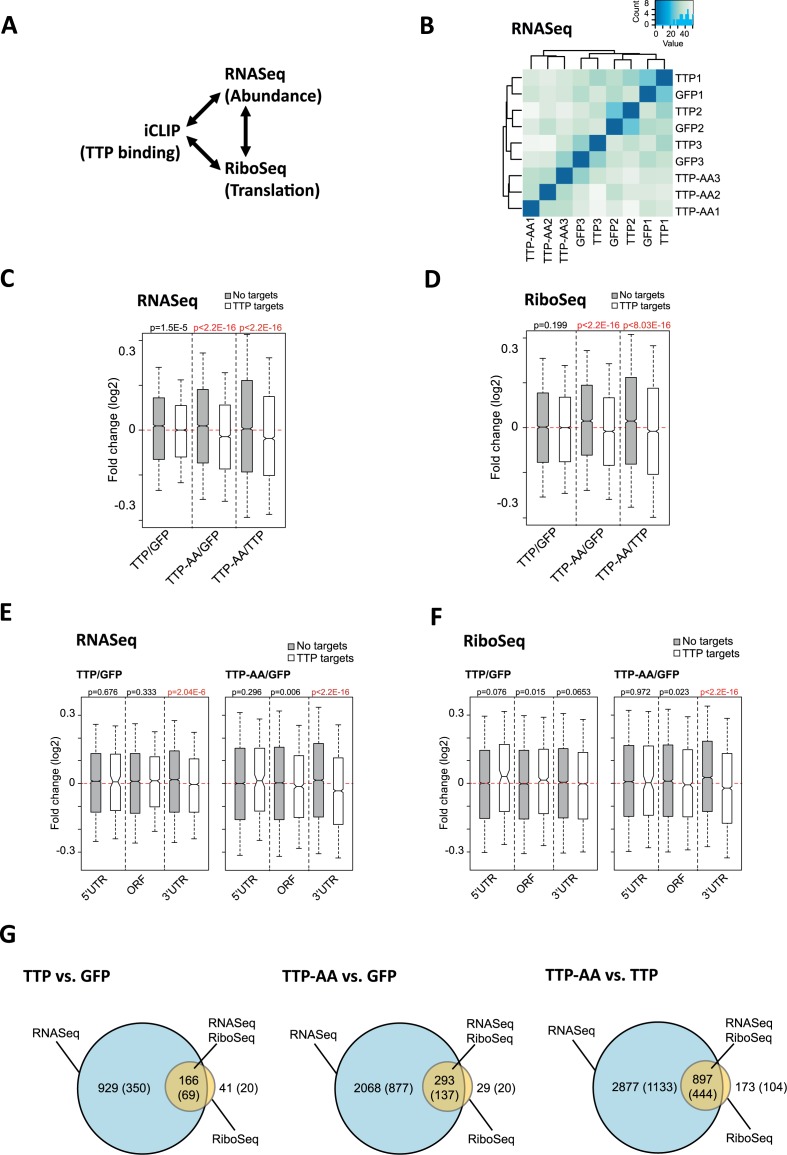
Correlation of iCLIP, mRNA abundance (RNASeq) and translation (RiboSeq) to identify global consequences of TTP- and TTP-AA-binding for the myeloid transcriptome. (**A**) Scheme for the correlations made between RNA levels or abundance (RNASeq), ribosomal occupation of mRNAs (RibosSeq) and the iCLIP data. RNASeq and RiboSeq measurements were performed in triplicate for each GFP-protein. See Supplementary Table S1 for number of replicates and conditions. (**B**) Cluster analysis for the RNASeq samples. Three replicates of cells each expressing GFP/EV, GFP–TTP or GFP–TTP-AA following 6 h Dox/1h LPS, are numbered 1, 2 and 3. (**C** and **D**) Overall changes in mRNA abundance (RNASeq (c)) and mRNA translation (RiboSeq (d)) between the indicated genotypes comparing the following TTP targets: iCLIP targets (white boxes) and non-bound mRNAs (gray boxes) are shown as a kernel density distribution in boxplots. A Wilcoxon rank sum test with continuity correction was used to determine the *P*-values shown for each comparison. Statistically significant differences in abundance and translation between targets and non-targets are indicated in red. For each genotype n = 3 sequencing libraries were analyzed and the mean of each group is shown. The red lines indicate for *y* = 0. Highly statistically significant differences are indicated in red. (**E** and **F**) For the comparisons between TTP/GFP and TTP-AA/GFP changed targets obtained from (C) and (D) were assigned to the mRNA region (5′UTR, ORF and 3′UTR) leading to detailed (E) RNASeq and (F) RiboSeq blots. A Wilcoxon rank sum test with continuity correction was used to determine the *P*-values shown for every comparison. The red lines indicate for *y* = 0. Highly statistically significant differences in abundance (E) and translation (F) between targets (white boxes) and non-targets (gray boxes) are indicated in red and could only be detected for targets where TTP binds in the 3′UTR. (**G**) The overlap of DE genes from RNASeq and RiboSeq experiments is visualized by Venn diagrams for the different comparisons. The number of genes for the RNASeq (changes in mRNA abundance), RiboSeq (changes in translation) and RNASeq/RiboSeq (changes in abundance and translation) are given together with the number of iCLIP targets in each group (in brackets).

Next, we analyzed the functional consequences of TTP binding to its RNA targets in a global manner by relating iCLIP data to the global analysis of mRNA abundance and translation after 1h of LPS-treatment, as measured by RNASeq and ribosome footprinting (termed RiboSeq (51)), respectively. Figure 2A and Supplementary Table S2 summarize the high-throughput sequencing approaches, their conditions and replicate numbers performed in this study. Cluster analysis of RNASeq data showed a distinct difference in the transcriptome of macrophages expressing mutant TTP-AA compared to those expressing wild-type TTP (Figure 2B). In contrast, samples from GFP-only expressing TTP-deficient macrophages and GFP–TTP expressing macrophages did not cluster separately as independent groups (Figure 2B). This suggested a greater overall similarity of their transcriptomes and strongly supports the notion that, following 1h of stimulation with LPS, inactivation of wild-type TTP by phosphorylation liberates mRNA targets from TTP-dependent regulation.

Similarly, global analysis of the change in mRNA abundance and translation in TTP-deficient macrophages (GFP controls) versus GFP–TTP expressing macrophages showed no differences (Figure 2C and D). By contrast, mRNA abundance and translation of TTP target mRNAs was significantly decreased in macrophages expressing mutant GFP–TTP-AA, compared to both GFP-only and GFP–TTP expressing macrophages (Figure 2C and D). This reveals an overall dependency of the de-repression of translation on phosphorylation of TTP by MK2. We observed that the global effect of the TTP-AA mutant on mRNA abundance and translation was independent of the abundance of the targets (data not shown). Importantly, global changes in the abundance and translation of TTP mRNA targets could be linked to TTP and TTP-AA binding to the 3′UTR, but not to the 5′UTR or the ORF of the mRNAs (Figure 2E for abundance and 2F for translation). This indicated that expression of a non-MK2-phosphorylatable TTP mutant in macrophages reduced translation and/or abundance of its 3′UTR-bound mRNAs in a global manner.

Differential expression (DE) analysis revealed a considerable overlap between mRNAs that are altered in abundance and in translation, in all pair-wise comparisons performed between the three conditions (GFP-only, GFP–TTP and GFP–TTP-AA expressing macrophages). The complete differentially expressed gene analyses for RNASeq and RiboSeq data can be found in the Supplementary Data Sets 2 and 3 and is summarized in Table 1. Generally we observed the following major findings: (i) Changes in mRNA abundance were more frequent (e.g. DE RNASeq, AA versus TTP, 3774 genes) than in translation (e.g. DE RiboSeq, AA versus TTP, 1070 genes) and ∼40% of the altered mRNAs were found to be TTP targets in our iCLIP experiments (Figure 2G, Table 1 and Supplementary Data Set 4). (ii) The comparison between TTP and TTP-AA (right column of Table 1) indicated that TTP phosphorylation is a landmark for post-transcriptional regulation of a large group of mRNAs at the level of abundance (DE RNASeq only, AA versus TTP, 2877 genes, 72.9% of all 3947 differentially expressed genes), while a smaller group of mRNAs is changed in both, translation and abundance (DE RNASeq + DE RiboSeq, AA versus TTP, 897 genes, 22.7%), and only a small fraction of targets is exclusively regulated at the level of RNA translation (DE RiboSeq only, AA versus TTP, 173 genes, 4.4%, Table 1). (iii) Of the 1681 iCLIP targets differentially expressed between TTP-AA and TTP rescued macrophages, 1133 (67.4%) are exclusively regulated in their abundance (e.g. Mdm2), 444 (26.4%) are regulated at the level of abundance and translation (e.g. Ier3) and 104 (6.2%) are exclusively regulated in their translation (e.g. Ccl3).

**Table 1.** Differential gene expression (DE) analyses of RNASeq and RiboSeq experiments. Cells were stimulated for a total of 6 h with doxycycline combined with 1 h LPS (6 h Dox/1h LPS) for the last hour

DE analysis	TTP versus GFP	TTP-AA versus GFP	TTP-AA versus TTP
**DE genes [#]**	**1137**	**2390**	**3947**
RNASeq [# of DE genes (% of all)]	1095 (96.3%)	2361 (98.7%)	3774 (95.6%)
RiboSeq [# of DE genes (% of all)]	208 (18.3%)	322 (13.5%)	1070 (27.1%)
RNASeq only [# of DE genes (% of all)]	929 (81.7%)	2068 (86.5%)	2877 (72.9%)
RNASeq and RiboSeq [# of DE genes (% of all)]	166 (14.6%)	293 (12.3%)	897 (22.7%)
RiboSeq only [# of DE genes (% of all)]	41 (3.6%)	29 (1.2%)	173 (4.4%)
**DE and iCLIP [#/%]**	**435 (38.3%)**	**1034 (43.3%)**	**1681 (42.6%)**
DE and iCLIP target in RNASeq [#/%]	419 (96.3%)	1014 (98.1%)	1577 (93.8%)
DE and iCLIP target in RiboSeq [#/%]	90 (20.1%)	157 (15.2%)	548 (34.7%)
DE and iCLIP target in RNASeq only [#/%]	350 (80.5%)	877 (84.8%)	1133 (67.4%)
DE and iCLIP target in both [#/%]	69 (15.9%)	137 (13.3%)	444 (26.4%)
DE and iCLIP target in RiboSeq only [#/%]	20 (4.6%)	20 (1.9%)	104 (6.2%)

The number of differentially expressed (DE) genes in each group, their overlap within the two groups and their overlap with the iCLIP occurrences were calculated and are given as absolute numbers and percentages in relation to all DE genes and all DE iCLIP targets, respectively.

### Major TTP targets in LPS-stimulated macrophages and their regulation by phosphorylation of TTP

The ability of TTP to regulate the pro-inflammatory response by affecting TNF/NF-κB-mediated signaling, chemokine as well as MAPK signaling has been suggested (24). Among the top 25 TTP mRNA targets (ranked by the overall number of cross-links in the 3′UTR) (Table 2), well known TTP-targets like TNF, C-X-C motif ligand 2 (Cxcl2) or C-C motif ligand chemokine 3 (Ccl3) were identified and serve as positive controls to validate our analysis (a comprehensive list of known TTP targets can be found in reference (4)). However, novel or poorly characterized targets, such as the E3 ubiquitin-protein ligase Mdm2, tumor necrosis factor alpha-induced protein 3 (Tnfaip3, A20), C–C motif ligand 4 (Ccl4), growth differentiation factor 15 (Gdf15) or C–X–C motif ligand 10 (Cxcl10) are also part of the top list (Table 2, marked in bold). Among the top targets of TTP, we observe an over-representation of cytokines/chemokines, including Cxcl10 and Gdf15. mRNAs encoding feedback inhibitors of the inflammatory response, such as TNF, Ier3 and Dusp1 (14), display significantly high numbers of crosslinks in the 3′UTR (Table 2, selected targets). Most, but not all of the top targets contain one or more ideal AUUUA-motif repeat(s) in their 3′UTR (Table 2, middle column). The transcripts without obvious AREs are indicated in italics in Table 2.

**Table 2.** List of the top 3′UTR target mRNAs and other selected TTP iCLIP targets of the GFP–TTP/GFP–TTP-AA group upon 1h LPS stimulation in combination with a total of 6 h doxycycline induction

**Top 3**′**UTR targets – 6 h Dox/1h LPS**				
Target mRNA	3′UTR length [nt]	AUUUA clusters	cDNA counts	TTP–AA/TTP counts
Cxcl2	705	11	2771	9.96
Tnf	762	8	2412	2.30
**Mdm2**	**1221**	**8**	**1716**	**10.87**
*Actb*	*681*	*0*	*1431*	*3.59*
Ccl3	407	5	1187	3.84
Ccl2	275	1	993	11.27
**Ccl4**	**302**	**2**	**956**	**13.22**
**Ybx3**	**577**	**2**	**946**	**5.98**
**Tnfaip3**	**6184**	**5**	**753**	**12.07**
Nfkbia	509	2	729	11.22
**Lpl**	**2644**	**5**	**640**	**6.00**
**Spp1**	**432**	**2**	**568**	**4.71**
**Cxcl10**	**1052**	**3**	**567**	**42.23**
Cdkn1a	1329	3	544	10.61
**Nlrp3**	**693**	**5**	**538**	**1.74**
*Lars2*	*986*	*0*	*516*	*n.d*.
Plau	938	2	505	16.38
Pfn1	272	1	485	5.90
**B2m**	**448**	**1**	**467**	**15.74**
*Sdc4*	*1653*	*0*	*465*	*7.56*
**Tnfsf9**	**253**	**2**	**462**	**16.50**
**Icam1**	**851**	**4**	**427**	**19.00**
*Tmsb4x*	*406*	*0*	*418*	*14.52*
**Nfkbiz**	**1353**	**4**	**403**	**31.25**
**Gdf15**	**148**	**5**	**384**	**3.03**
**Selected targets**				
Ier3	601	5	255	2.3
Dusp1	696	4	291	2.5

**Figure 3.**
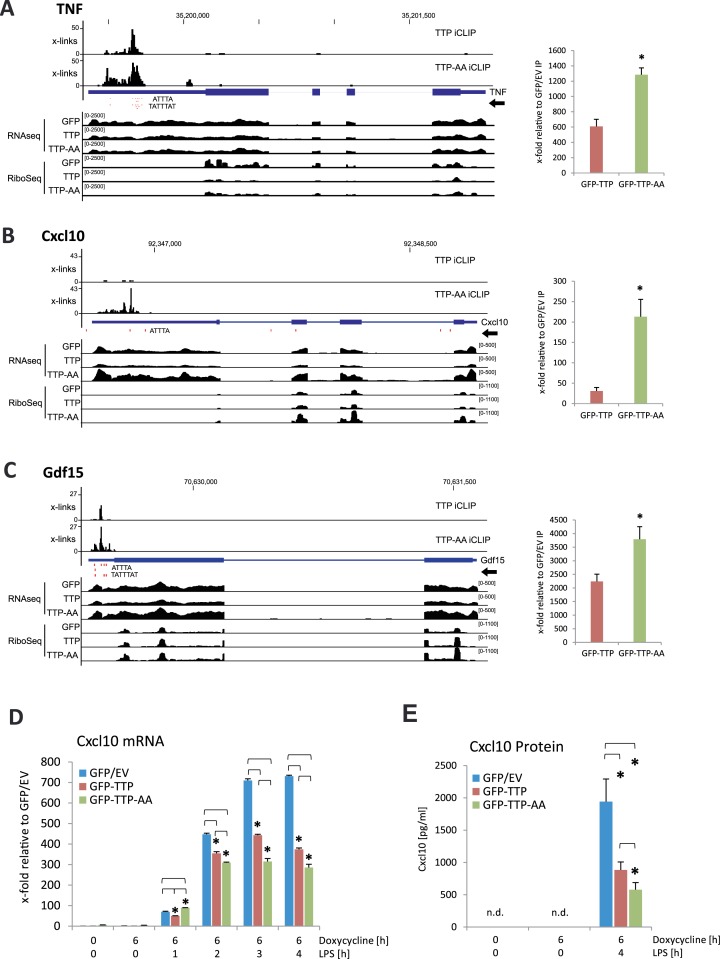
Examples of established and novel TTP targets identified by iCLIP and their validation by RNA-IP and expression analysis. (**A–C**) Genomic tracks for TNF (A), Cxcl10 (B) and Gdf15 (C) are shown, containing information for iCLIP, RNASeq and RiboSeq for GFP-, GFP–TTP- and GFP–TTP-AA-expressing cells. ATTTA and TATTTAT repeats are indicated as red dots below the iCLIP tracks. Dimensions are indicated by the numbers in brackets for each track. At the right of the tracks, RNA-IPs for GFP–TTP and GFP–TTP-AA are shown in relation to the GFP/EV control IP. The asterisk (*) indicates *P*-values ≤ 0.05 for GFP–TTP-AA IP compared to GFP–TTP IP. (**D**) Cxcl10 mRNA level in the three different cell lines were determined by qPCR. (**E**) Cxcl10 protein levels were determined by ELISA for the indicated stimulatory conditions. 1 × 10^4^ cells were seeded in triplicates in a 96-well plate. After attachment of the cells, stimulation was started and Cxcl10 protein in supernatants was measured. The asterisk (*) indicates *P*-values ≤ 0.03; n.d.: not detectable.

The schematic representation of the combined iCLIP, RNASeq and RiboSeq analyses of a known (TNF) and two novel TTP-targets (Cxcl10 and Gdf15) are shown in Figure 3A–C. By comparing the iCLIP crosslinks of TTP and TTP-AA in these genomic tracks, a significantly higher binding of TTP-AA to these transcripts is apparent. For all top 3′UTR targets binding of GFP–TTP-AA to target 3′UTRs was more frequent compared to GFP–TTP (Table 2, right column). This could reflect the phosphorylation-dependent release of TTP, but not of TTP-AA, from the AREs after 1h of LPS stimulation.

The association of TNF, Cxcl10 and Gdf15 mRNA to TTP was additionally confirmed by RNA-immunoprecipitation (RNA-IP) experiments after 1 h of LPS treatment (Figure 3A–C and Supplementary Figure S4a). The relative enrichment of mRNAs in GFP–TTP- and GFP–TTP-AA-IP compared to their presence in control GFP-IP is shown in the right panels of Figure 3A–C. Enrichments (20- to 4000-fold) were obtained in the RNA-IP experiments for all three mRNAs confirming the TTP-binding detected in iCLIP. In all cases, GFP–TTP-AA precipitation recovered significantly more transcript than GFP–TTP, again indicating stronger binding of the non-phosphorylated TTP. In addition we also analyzed the functional properties of target 3′UTRs in luciferase reporter experiments. The insertion of the 3′UTRs of Cxcl10 and Gdf15 mRNA into the reporter plasmid strongly reduced reporter activity (Supplementary Figure S4b). Co-expression of GFP–TTP or GFP–TTP-AA led to a further reduced reporter activity (Supplementary Figure S4c). Polysome analyses showed that TTP and TTP-AA not only regulate the association of TNF mRNA, but also of Gdf15 mRNA to actively translating ribosomes. Association of Cxcl10 and of β-Actin mRNA remained unaffected (Supplementary Figure S5a-c). The mRNA of β-Actin was in the list of top iCLIP targets, but has no ARE in its 3′UTR (Table 2) and no effect on its abundance was detected after TTP-expression (data not shown). We therefore considered β-Actin mRNA as a negative control for TTP binding.

Since the novel TTP-target Cxcl10 is a secreted cytokine of the chemokine family (52), we further monitored its TTP-dependent expression. The results indicated a strong effect of both TTP and TTP-AA on Cxcl10 mRNA level (Figure 3D) and protein synthesis (Figure 3E) showing that TTP binding to AREs in the 3′UTR of Cxcl10 is influencing the post-transcriptional regulation of this cytokine only at the level of mRNA abundance, because we did not observe obvious changes in polysome association of its mRNA (Supplementary Figure S5c).

### Phosphorylation-dependent TTP inactivation is required for NF-κB-signal transduction

**Figure 4.**
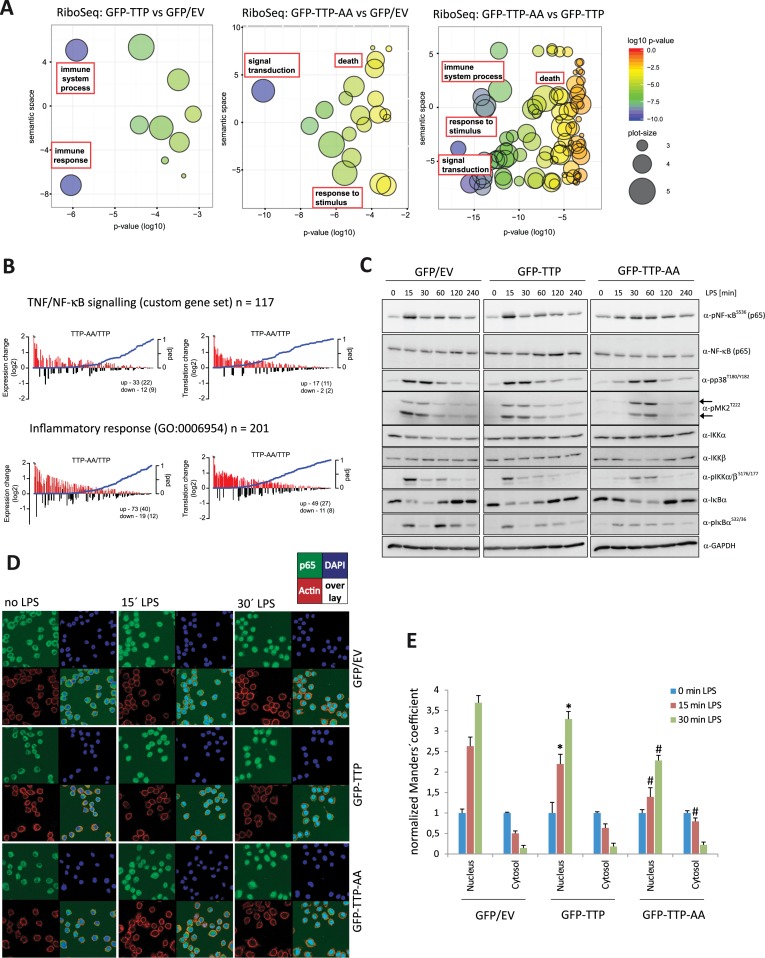
Gene ontology analysis reveals a role of TTP in signal transduction processes including NF-κB -signaling. (**A**) Differentially expressed genes from RiboSeq data comparing different samples were used to perform gene ontology (GO) analyses using the GOrilla tool (47). To remove redundant terms and to visualize the results the REVIGO tool (85) was used. The y-axis (semantic space), that quantifies similarities of word meanings. The color-coded log10 *P*-values for each GO term are given on the x-axis. Circle sizes correspond to the frequency of similar GO terms summarized in the specific GO term (see legend on the right). Enriched GO terms connected to enriched pathways (Table 3) are highlighted in red rectangles. (**B**) A custom gene set comprised of 117 TNF-/NF-κB-signaling-related genes and the ‘inflammatory response’ (GO:0006954) were enriched for TTP-AA vs. TTP cell mRNA expression (left plot) and translation (right plot). Log2-fold changes in expression and translation of each regulated gene are plotted with the corresponding adjusted *P*-values (padj) of the changes. Numbers indicate increased or decreased genes. The associated numbers in brackets show the number of iCLIP targets in each corresponding group. (**C**) Components of the NF-κB-signaling pathway were analyzed in western blot in macrophages upon 6 h of doxycycline-stimulation prior to stimulation with LPS for the indicated times. In addition, the phosphorylation of p38^MAPK^ at T180/T182 and MK2 at T222 (double band pattern indicated by arrows) were monitored as controls for LPS-mediated TLR4-activation. Quantifications of these western blots are given in Supplementary Figure S6. (**D**) Examples of images used for the microscopic analysis of NF-κB localization (green) in the indicated cell types and upon different times of LPS stimulation. Nuclei (blue) and F-actin (red) were co-stained with DAPI and Phalloidin, respectively. An overlay of all three channels is added to each panel. (**E**) Quantitative analysis of the images exemplified in (D) to compare the increase of nuclear and decrease of cytosolic NF-κB localization upon LPS stimulation. For each analysis and each genotype, unstimulated cells were used as a reference. Nuclear localization was assessed by comparing with DAPI and cytosolic localization with F-actin co-staining (see experimental procedures for details). The hashtag (#) indicates statistical significance with *P*-values ≤ 0.05 comparing GFP–TTP-AA to GFP/EV and GFP–TTP cells, whereas the asterisk (*) indicates for significance comparing GFP–TTP with GFP/EV cells.

Next, we performed gene ontology (GO) analyses using the DE RiboSeq datasets to identify TTP-dependent translational RNA regulons. The analysis revealed commonly regulated groups of transcripts based on the biological process they are involved in (Table 3). After removal of redundant GO terms, we identified ‘immune response’, ‘cell death’, ‘response to stimulus’ and ‘signal transduction’ as terms designating mRNAs commonly regulated by TTP-phosphorylation in LPS-stimulated macrophages (Figure 4A). When differentially expressed genes from RNASeq experiments (Supplementary Table S3) or the list of TTP-bound mRNAs (data not shown) were used, similarly enriched pathways as highlighted in Figure 4A were uncovered. The gene signature of differentially expressed and translated iCLIP target mRNAs was associated with TNF/NF-κB-signaling (Supplementary Data Set 5) and the inflammatory response. Interestingly, GFP–TTP-AA expressing cells showed an increase in mRNA abundance and translation of mRNAs associated to TNF/NF-κB-signaling when compared to GFP–TTP cells at 1h LPS stimulation (Figure 4B). Approximately 50% of the DE genes associated with these pathways were also identified as TTP targets in our iCLIP experiments, strengthening the notion that TTP acts as a global regulator of the abundance and translation of mRNAs involved in signal transduction during the inflammatory response. By contrast, no significant changes in the inflammatory response gene signature were found when comparing TTP-expressing cells and GFP control cells (data not shown), again suggesting a similarity between macrophages that lack TTP and those that contain wild-type TTP during the early stages of LPS-dependent cell activation.

**Table 3.** Enriched pathways determined by WebGestalt for the differentially expressed genes obtained from the RiboSeq data for cells expressing GFP/EV (GFP), GFP–TTP (TTP) or GFP–TTP-AA (AA) upon 6 h Dox/1 h LPS treatment

**1. RiboSeq TTP versus GFP**			
Pathway Name	# reference genes	#DE genes/# iCLIP targets	adjusted *P*-value
PluriNetWork	292	8/7	3.39E-5
mRNA processing	483	9/6	9.93E-5
Focal Adhesion	186	5/4	0.0006
**2. RiboSeq TTP-AA vs. GFP**			
**Apoptosis**	93	6/4	0.0001
Myometrial Relaxation and Contraction Pathways	158	7/3	0.0001
miRNA regulation of DNA Damage Response	69	5/2	0.0001
Calcium Regulation in the Cardiac Cell	152	7/2	0.0001
Glycolysis and Gluconeogenesis	51	5/3	0.0001
**Chemokine signaling pathway**	186	6/1	0.0018
**T Cell Receptor Signaling Pathway**	143	5/5	0.0019
**Wnt Signaling Pathway NetPath**	141	5/1	0.0019
PluriNetWork	292	7/5	0.0019
**TNF-alpha NF-kB Signaling Pathway**	215	6/4	0.0019
**3. RiboSeq TTP-AA vs. TTP**			
mRNA processing	483	42/28	7.5E-15
PluriNetWork	292	31/23	1.68E-13
**TNF-alpha NF-kB Signaling Pathway**	215	26/18	8.01E-13
**Chemokine signaling pathway**	186	21/10	6.24E-10
Macrophage markers	10	7/2	9.22E-10
**Apoptosis**	93	15/9	1.59E-9
Focal Adhesion	186	18/14	1.02E-7
Adipogenesis	133	15/10	1.88E-7
Myometrial Relaxation and Contraction Pathways	158	16/11	2.7E-7
**G Protein Signaling Pathways**	94	12/7	6.88E-7

Next, we validated potential alterations in the TNF/NF-κB-signaling pathway, the inflammatory response and the prolonged activation of p38^MAPK^ and MK2 in LPS-induced signaling (Figure 4C). As expected from our pathway enrichment analysis, we did not observe differences in cellular signal transduction between TTP-deficient and TTP-expressing macrophages. However, macrophages expressing the TTP-AA mutant displayed profound alterations in both NF-κB- and p38^MAPK^/MK2-signaling. Phosphorylation of the p38^MAPK^-signaling proteins was significantly delayed (Figure 4C, and Figure 1C), whereas phosphorylation of NF-κB (p65) was decreased, but prolonged (Figure 4C, and quantified in Supplementary Figure S6a). Upstream components of the NF-κB-signaling module such as IKKα/β and IκBα showed a similarly delayed and decreased activation pattern (Figure 4C). As phosphorylation of NF-κB ultimately leads to its nuclear translocation and transcriptional activation of responsive genes (summarized in (53)), we investigated whether the delayed phosphorylation kinetics also results in impaired nuclear translocation. Nuclear NF-κB becomes detectable shortly after LPS stimulation in all of the three cell types (Supplementary Figure S6b). By calculating the nuclear to cytosolic ratio of NF-κB protein at each time point, we detected a decreased and delayed translocation of p65/ NF-κB in both, TTP-AA- and TTP-expressing cells, compared to GFP-only cells (Supplementary Figure S6c). To validate this effect more quantitatively, we quantified fluorescence signals in cytosolic and nuclear compartments (Figure 4D and E). Expression of GFP–TTP and GFP–TTP-AA slowed down NF-κB nuclear import and we observed an increased effect in GFP–TTP-AA cells, which was in line with the observations made by cellular fractionation and immunoblotting (Figure 4C). The delayed NF-κB translocation is paralleled by global changes in cytokine expression in these cells (Supplementary Figure S7). This may at least in part reflect functional consequences of the alteration in NF-κB signaling. Cluster analyses of both RNASeq and RiboSeq data showed alterations in cytokine mRNA levels in cells expressing the mutant TTP-AA, as compared to the other two cell types. Discrete clustering of the three individual replicates of TTP-AA cells (AA1-3) was observed in these analyses, whereas replicates of GFP- and TTP-expressing cells failed to cluster based on the expression of wild type TTP (Supplementary Figure S7).

In summary, the predictions from our high-throughput sequencing data analysis, indicating that MK2-dependent inactivation of TTP is required for signal transduction, were validated. By phospho-kinetic analyses we showed that in macrophages the post-translational regulation of TTP is required for proper LPS-stimulated NF-κB phosphorylation and for its translocation to the nucleus to trigger the cytokine response.

### TTP limits the expression of feedback inhibitors

**Figure 5.**
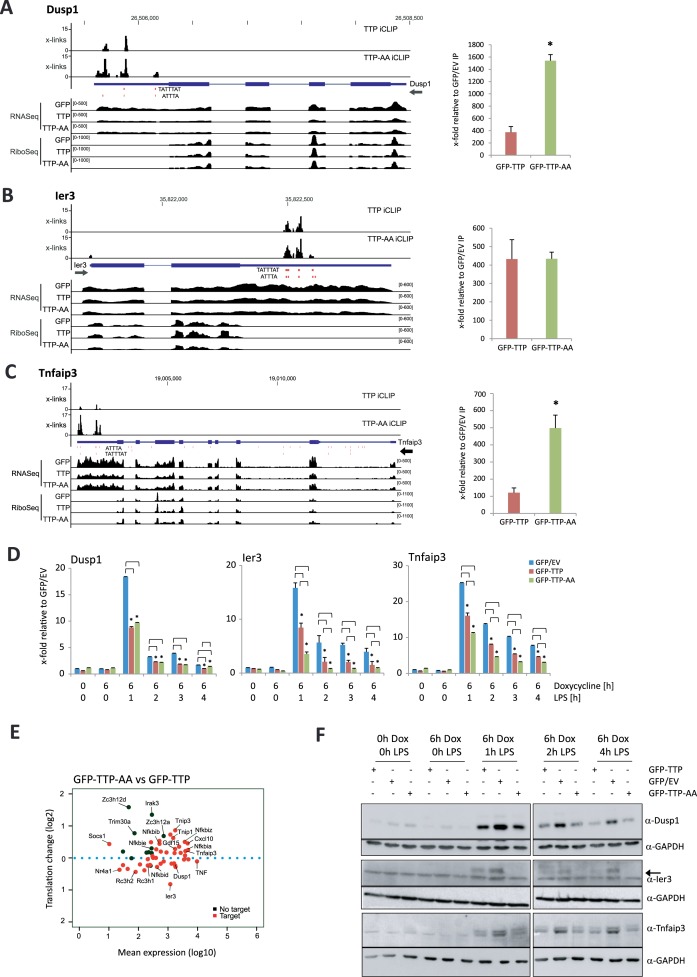
Deregulation of feedback inhibitors of inflammation through TTP and TTP-AA. (**A–C**) Genomic tracks for the feedback inhibitors Dusp1 (A), Ier3 (B) and Tnfaip3 (C) containing information for iCLIP, RNASeq and RiboSeq data for GFP-, GFP–TTP- and GFP–TTP-AA-expressing cells are shown in the format as introduced in Figure 3. ATTTA and TATTTAT repeats are indicated below the iCLIP tracks. Dimensions are indicated by the numbers in brackets for each track. The graph to the right of each track shows the RNA-IPs for GFP–TTP and GFP–TTP-AA in relation to the GFP/EV Control IP. Asterisks (*) indicate *P*-values ≤ 0.05 for GFP–TTP-AA IP compared to GFP–TTP IP. (**D**) Relative mRNA levels of the indicated transcripts were determined by qPCR upon different stimulations. The control conditions of GFP/EV-expressing cells were set to 1. Fold changes were calculated in relation to this condition. (**E**) Log_2_-fold changes in translation versus changes in mean expression (log_10_) for a set of feedback inhibitors of the inflammatory response (14) and selected other mRNAs in GFP–TTP-AA- versus GFP–TTP-expressing cells. The position of individual transcripts is marked. TTP-bound iCLIP target mRNAs are shown as red dots, whereas non-targets are black. (**F**) Levels of the Dusp1-, Ier3- and Tnfaip3-protein were determined by western blot in the TTP BMDM cell lines upon different stimulations. GAPDH served as a loading control. Quantifications of western blots are given in Supplementary Figure S8.

The identified target mRNAs of TTP, coding for feedback inhibitors of the inflammatory response such as Tnfaip3/A20 (17), Ier3 (14,54–55) or Dusp1 (56), as well as TNF itself (summarized in (57,58)), are closely connected to NF-κB-activation. Thus, we hypothesized that the expression and translation of mRNAs encoding feedback inhibitors of the inflammatory response were controlled by TTP. Genomic tracks for Tnfaip3, Ier3 and Dusp1 show TTP and TTP-AA binding to AREs within their 3′UTRs (Figure 5a–c left), which is confirmed by RNA-IP (Figure 5A–C right). Similar to TNF, Cxcl10 and Gdf15 mRNA (Figure 3A–C), we observed more crosslinks of TTP-AA for Dusp1, Ier3 and Tnfaip3 representing a potential stronger binding of mutant TTP compared to the wild-type protein. This conclusion is further supported by RNA-IPs capturing these mRNAs more effectively with GFP–TTP-AA. Analysis of the mRNA levels of the targets Dusp1, Ier3, and Tnfaip3 by RT-qPCR confirmed that TTP and TTP-AA expression reduced mRNA abundance of all transcripts upon macrophage stimulation with LPS (Figure 5D). Whereas wild-type and mutant TTP affected Dusp1 mRNA to similar extend, TTP-AA had greater impact on Ier3 and Tnfaip3 mRNA levels. A similar reduction was observed for the TTP's *bona fide* target TNF, that itself is a member of the feedback inhibitor group (Supplementary Figure S1f). To demonstrate the link TTP of binding to decreased expression, the 3′UTR of Tnfaip3 mRNA was inserted into a luciferase reporter. This led to decreased luciferase expression (Supplementary Figure S4d). Further TTP-dependent reduction of luciferase expression was observed, without any additional effects of the TTP-AA mutant (Supplementary Figure S4e).

Analysis of mRNA translation of both feedback inhibitors of the inflammatory response and components of the NF-κB-signal transduction pathway, such as Nfkbia, Nfkbib, Nfkbid, Nfkbie and Nfkbiz, revealed that many of these TTP-targeted mRNAs were translationally deregulated at 1h of LPS stimulation in macrophages expressing the mutant TTP-AA (Figure 5E). Further analysis of protein abundance of feedback inhibitors Dusp1, Ier3, Tnfaip3 and TNF by western blot revealed that expression of TTP and TTP-AA reduced the level of these proteins (Figure 5f, Supplementary Figure S1h). For TNF and Ier3 we observed reduced protein levels in TTP-AA cells, when compared to wild-type TTP-expressing cells (Figure 5F, Supplementary Figures S1 and S8). Of note, the protein level reduction in macrophages expressing TTP or TTP-AA was not only due to a potential destabilization of TTP-targeted mRNAs (Figure 5D), but could also be explained by less effective polysome association of the these target mRNAs (Supplementary Figure S5b and d). The strongest translational repression was observed for Ier3 and TNF at all times of LPS stimulation and mild effects were observed for Dusp1 and Tnfaip3 at different times of LPS stimulation (Supplementary Figure S5d). Taken together, our data suggest that TTP is required for the precise modulation of the pro-inflammatory response to LPS. It functions by directly binding to the 3′UTR of components of the TNF/NF-κB-mediated signaling and post-transcriptional regulation of feedback inhibitors of the inflammatory response, such as Dusp1, Ier3, Tnfaip3 and TNF. Of note, this TTP-dependent regulation is not specific for macrophages and was also confirmed in fibroblasts (Supplementary Figure S9).

### TTP phosphorylation by MK2 prevents cell death

**Figure 6.**
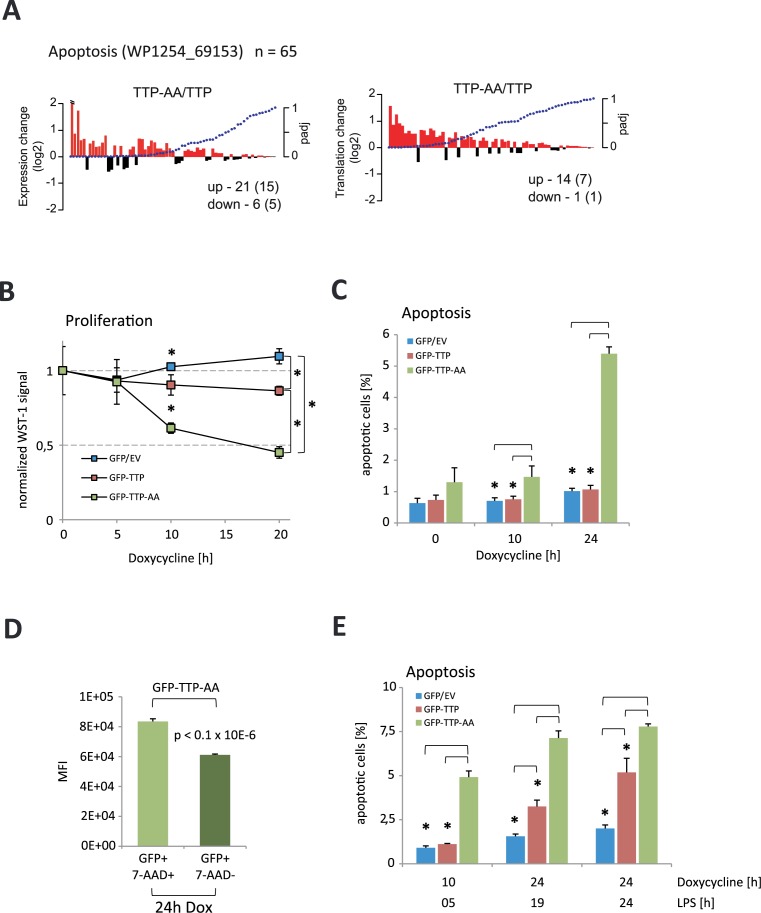
TTP-AA-expression limits proliferation and increases cell death. (**A**) The 65 genes of the enriched Wiki pathway ‘Apoptosis’ (WP1254_69153) were analyzed for changes in their expression (left panel) and translation (right panel) comparing GFP–TTP-AA to GFP–TTP cells. Log2-fold changes are plotted against the adjusted *P*-values of the changes. The numbers of up- and down-regulated genes are given (number of iCLIP targets in brackets). (**B**) Cell proliferation of macrophages after induction of GFP, GFP–TTP and GFP–TTP-AA expression by doxycycline treatment. Non-doxycycline treated cells of each genotype were used for normalization of signals. The mean of all three measurements is shown and standard deviations are included. The asterisk (*) indicates *P*-values ≤ 0.05. (**C**) Percentages of apoptotic cells upon long-term expression of GFP-fusion proteins were determined. The mean and standard deviation of three independent experiments are shown. The asterisk (*) corresponds to *P* < 0.03. (**D**) Mean fluorescence intensities (MFI) of GFP fluorescence in apoptotic (7-AAD+) and non-apoptotic (7-AAD-) GFP–TTP-AA-expressing cells. *P* < 0.1×10^−6^ (unpaired *t*-test). (**E**) Cell death after induction of GFP/GFP–TTP/GFP–TTP-AA expression and LPS stimulation for different times. The mean and standard deviation of three independent experiments s are shown. The asterisk (*) corresponds to *P* < 0.005.

Deregulation of NF-κB-signaling is associated with altered inflammatory responses and macrophage cell death (summarized in (53,59)). Our gene set enrichment analysis of changes between TTP-AA- and TTP-expressing macrophages highlighted cell death as a pathway that was deregulated. Characterization of a set of 65 genes related to apoptosis showed that 21 genes (including 15 iCLIP targets) were differentially increased at the level of mRNA abundance and 14 genes (seven iCLIP targets) showed enhanced translation in TTP-AA macrophages compared to TTP macrophages (DESeq2, padj<0.05, Figure 6A). 11 out of these 14 genes showed a significant change in both mRNA abundance and translation. Within this group of 11 genes we identified several pro-apoptotic genes, such as Bbc3, Pmaip1, Tnfrsf1a, Bak1 and Casp4 (DESeq2, padj<0.05, Figure 6A). Three genes with increased mRNA translation in TTP-AA expressing macrophages and with relation to cell death were the pro-apoptotic genes Bcl2l11, Cdkn2a and Fas. All these mRNAs, except Fas and Bak1, were identified as TTP targets in our iCLIP experiments suggesting that MK2-mediated modulation of TTP activity is involved in the control of cell survival.

Analysis of the growth and viability of TTP-rescued macrophages by a WST-1 assay showed that an artificial long-term induced expression of GFP–TTP only moderately reduced viability and proliferation of macrophages compared to GFP-only control cells (Figure 6B). Interestingly, in the same system the expression of GFP–TTP-AA was strongly anti-proliferative and reduced the viability of cells to 50% after 20h of doxycycline treatment (Figure 6B). This suggested a potential role for TTP in cell cycle regulation as proposed for the other two Zfp36 family members Zfp36l1 and Zfp36l2 (60). Analysis of apoptosis by 7-amino-actinomycin D (7-AAD) incorporation showed that induction of GFP–TTP-AA expression with doxycycline increased the number of apoptotic cells over time, whereas expression of GFP and GFP–TTP had no effect (Figure 6C). Quantitation of GFP mean fluorescence intensities in 7-AAD^+^ and 7-AAD^−^ cells demonstrated that expression of GFP–TTP-AA significantly correlated with cell death (Figure 6D). Finally, we also measured cell susceptibility to LPS-induced apoptosis. It was seen that GFP–TTP-AA expression significantly increased cell death compared to macrophages expressing GFP–TTP or GFP. Only TTP-deficient cells were protected against LPS-induced cell death for up to 24 hours (Figure 6e). Altogether, our results indicated that TTP phosphorylation by MK2 was required for TTP inactivation and preserved macrophages from cell death after LPS stimulation.

## DISCUSSION

**Figure 7.**
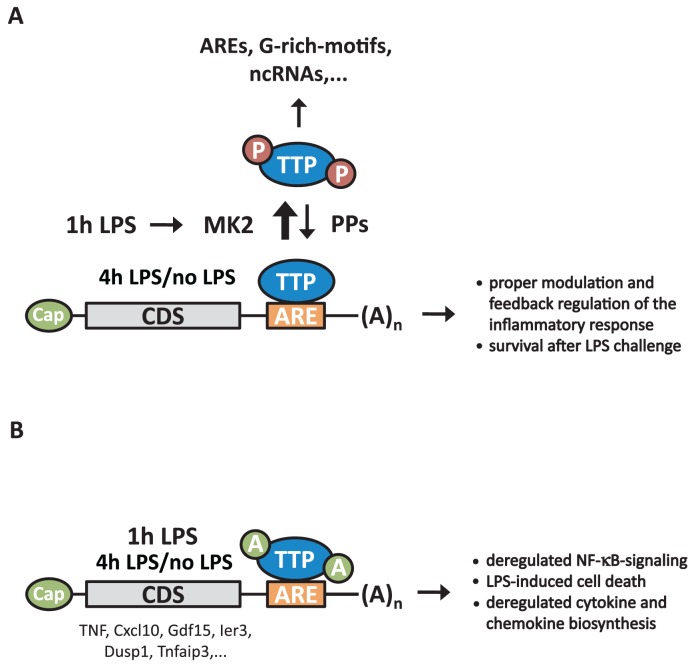
Schematic presentation of TTP's action as phosphorylation-triggered key regulator of feedback control of inflammation via NF-κB-regulating proteins, cytokines and chemokines. (**A**) Upon LPS-mediated TLR4-stimulation (1 h LPS), TTP is phosphorylated and thereby inactivated by MK2. As a consequence and as detected by iCLIP analyses, binding to AREs within 3′UTRs is decreased, compared to non-phosphorylated TTP (no LPS) or at later time points when TTP phosphorylation decreases (4 h LPS). Under physiological conditions, phosphatases (PPs), such as PP2A, are responsible for dephosphorylation of TTP after prolonged LPS challenge, restoring TTP's binding to AREs to mediate post-transcriptional control of associated mRNAs. An enhanced association of phospho-TTP to ncRNAs was also detected without determining its physiological role. (**B**) Absence of MK2-mediated TTP-phosphorylation at serines S52/S178 in TTP-AA cells results in enhanced binding to AREs in the 3′UTRs of mRNAs coding for stimulators and feedback regulators of inflammation especially after 1h of LPS stimulation. This results in their post-transcriptional regulation at the level of mRNA stability and translation. As a direct output we detected a stronger suppression of stimulatory cytokines and chemokines, such as TNF, Cxcl10 and Gdf15, and of negative feedback inhibitors, such as Ier3, Dusp1 and Tnfaip3, in these cells, compared to wild-type TTP-expressing cells. In addition, several proteins coding for NF-κB-regulators are affected post-transcriptionally. Together this results in deregulated NF-κB-signaling and increased cell death in response to LPS. ARE: AU-rich element; Cap: 5′-7-methylguanosine cap; CDS: coding (DNA) sequence; (A)_*n*_: 3′-poly(A) tail.

The present study identifies *in vivo* targets of TTP and characterizes the function of TTP in LPS-stimulated macrophages, a TLR4-expressing cell type, where TTP shows major expression and plays a prominent regulatory role in the control of the inflammatory response (61). By using iCLIP to identify TTP targets and relating TTP-binding to TTP-dependent changes of mRNA abundance and translation, we provide a transcriptome-wide view of the regulation of TTP-bound RNAs. TTP is able to post-transcriptionally regulate its mRNA targets either by controlling RNA-stability and/or by regulating mRNA translation. TTP-action in coordinating a rapid and transient inflammatory response (62) is effectively regulated through its transient phosphorylation by MK2 (Figure 7A). Therefore, a mutant of TTP, which is not phosphorylated by MK2, displays enhanced binding to 3′UTRs of mRNA leading to deregulation of both the kinetics and the endpoints of the inflammatory response. These observations corroborate the findings reported for *in vitro* MK2-phosphorylated TTP (8). TTP not only regulates cytokine- and chemokine-coding transcripts, but also transcripts of essential feedback inhibitors of the inflammatory response. In the case of the TTP mutant, where phosphorylation-dependent regulation of TTP is defective, we observed a deregulation of NF-κB-signaling due to the post-transcriptional control of NF-κB-regulators. Similar to the results presented here, TTP-regulated RNAs identified in a MEF cell system and comparisons with analyses in human cells (see (31)) will enable us to further determine cell-type and stimulus-specific binding of TTP to RNAs.

Here we have identified 1112 mRNA targets of TTP and 3763 mRNA targets of TTP-AA in macrophages by iCLIP, which considerably extends the number of targets previously identified in a PAR-CLIP study that employed overexpressed FLAG/HA-tagged TTP in HEK293 cells (31), which do not carry TLR4 receptors and do not respond to LPS stimulation (14). Importantly, the TTP-AA mutant protein gave rise to more complex iCLIP libraries from a highly diverse pool of mRNA targets resulting in a higher abundance of unique cross-link sites compared to wild-type TTP. Both, TTP and TTP-AA displayed a strong association to AUUUA core motif-containing sequences in the 3′UTR of mRNAs, consistent with previous studies that determined the optimal TTP-binding sequence UUAUUUAUU (summarized in (4)). TTP-AA showed an even higher 3′UTR-enrichment than wild-type TTP and potentially a higher bias towards optimal AREs and its variants, symbolized as WWW(AUUUA)WWW (W could be A or U). These binding preferences reflect the ability of the individual zinc fingers of TTP to bind to UAUU, and exactly revealed the octamer UAUUUAUU that was identified as the binding motif of the tandem zinc finger domain in the TTP structure (63). For wild-type TTP we also identified exceptions of the classic binding motif. These G-rich sequences may reflect alternative binding modes of MK2-phosphorylated TTP. Additionally, our iCLIP data showed a rather uniform distribution of crosslinks within 3′UTRs. A linear increase of crosslinks was only observed within the first 100 nucleotides following the ORF-3′UTR junction. Apart from this, we found a random distribution of TTP-binding sites in 3′UTRs, which corresponds to the random distribution of AREs in this gene region (64). However, a clear enrichment of crosslinks was observed at the 3′UTR-intergenic region junction, indicating a preferential 3′UTR binding with maximal enrichment at about 70 nucleotides upstream of the ends of the 3′UTRs. It was previously proposed that TTP-binding is enriched near the very 3′ends of 3′UTRs (31), supporting the physiological role of TTP in deadenylation of mRNAs (11–13). Alternatively, this could also reflect a role of TTP in polyadenylation (65).

We found that iCLIP targets are regulated at the level of mRNA abundance and to a much lower extent at the level of translation. In particular, GFP–TTP-AA cells display the most prominent degree of regulation after 1 hour of LPS treatment indicating the physiological role of TTP phosphorylation in macrophages at a global level. This is also reflected by the loss of differences in 3′UTR binding without or with long-term LPS stimulation. Generally, for most changes in mRNA translation simultaneous changes in mRNA levels were observed, a finding that is consistent with previous studies of LPS-stimulated macrophages (14,66). Around 80% of all differentially expressed RiboSeq iCLIP targets are also differentially expressed in RNASeq analyses (TTP-AA vs. TTP, 448 of total 548). Most of the iCLIP targets are only regulated at the level of mRNA stability. This group of iCLIP target mRNAs is represented by around 94% (TTP-AA vs. TTP, 1592 of total 1692) of all differentially expressed genes that appear in the RNASeq-only group. Hence, next to the identification of its numerous targets we add a systemic view of the consequences of phosphorylation-regulated RNA-binding of TTP to target mRNAs.

In addition to known TTP targets that confirmed the experimental approach, other novel or poorly characterized targets of TTP such as Cxcl10 or Gdf15 were identified by iCLIP. Cxcl10 mRNA contains two pentameric AUUUA motifs in its 3′UTR that could serve as TTP-binding sites and yielded in a significant number of crosslinks in our analysis. Suppression of Cxcl10 mRNA and protein levels by TTP, confirmed that it is a true TTP target. Previously it was suggested that TTP-deficiency does not influence Cxcl10 mRNA levels (67). Another report documented p38^MAPK^/MK2-dependency of Cxcl10 levels, although this was found in the context of IFN-γ stimulated macrophages (52). The reason for these divergent results is not clear but could arise from analysis at different times and modes of stimulation. Further evidence is provided by the TTP^aa/aa^-mouse-model where both, Cxcl10 mRNA and protein were decreased *in vivo* (26). Similar to Cxcl10, Gdf15 was only poorly characterized as a target of TTP. Gdf15 was initially described as macrophage inhibitory cytokine (MIC-1) able to reduce TNF expression (68). Consequently it was reported that Gdf15 functions as an anti-inflammatory effector by reducing the recruitment of leukocytes to sites of inflammation. The authors suggested that Gdf15 could generally function in inflammatory diseases and cancer (69). We detected crosslinks of TTP and TTP-AA to AREs in the 3′UTR of Gdf15 mRNA. Taken together, these observations support our suggestion that Gdf15 and Cxcl10 are novel direct targets of TTP in LPS-stimulated macrophages.

Additionally, we observed the regulation of inflammatory feedback inhibitor mRNAs, such as those of Dusp1, Ier3 and Tnfaip3 (or A20) with Tnfaip3/A20 being a poorly characterized TTP-bound mRNA. Binding of TTP and TTP-AA to AREs within 3′UTRs of the mRNAs reduced their protein level through mRNA destabilization and partly through less effective translation. The dual-specific protein phosphatase Dusp1 has been described as an inhibitor of the p38^MAPK^-signaling module which is able to specifically dephosphorylate MAPKs (70). By inhibiting the activation of MAPKs such as p38s or ERKs (for example during pro-inflammatory conditions) Dusp1 is part of the feedback mechanism that limits inflammation (71). Deletion of Dusp1 results in deregulation of pro- and anti-inflammatory cytokine biosynthesis (15–16,18,21). The absence of Dusp1 prolongs MAPK-activation profiles and it leads to elevated TTP protein levels due to reduced dephosphorylation-dependent proteasomal degradation (71). TTP binding to the Dusp1 3′UTR has been suggested as mechanism of Dusp1 regulation in dendritic cells (29). The iCLIP data presented here revealed a direct binding of TTP and TTP-AA to the AREs within the Dusp1 3′UTR. Analysis of Dusp1 mRNA abundance by qPCR, protein expression and Dusp1 mRNA association with actively translating polysomes showed dependence on GFP–TTP and GFP–TTP-AA expression. This could contribute to the prolonged MAPK-activation profile detected in Figures 1C and 4C.

Ier3 is known to protect cells from apoptosis (55) and to interact with the NF-κB p65 subunit RelA (19,54,72). It has previously been identified as a target of TTP (27). Ier3 has been suggested to be regulated at the translational level in RAW264.7 cells and cells deficient in Ier3 are more susceptible to cell death upon LPS stimulation (14). The dependence of Ier3 regulation on TTP was not investigated. In this regard, our data indicated that TTP regulates Ier3 mRNA in a fashion very similar to TNF mRNA and leads to strong reduction of Ier3 protein also due to an interplay of translational control and stability regulation.

Tnfaip3/A20 was also identified as TTP target before, but the impact of TTP-binding was only analyzed with respect to destabilization of the mRNA (30). Tnfaip3/A20 is an ubiquitin-modifying enzyme and acts as a feedback regulator in inflammation and chronic-inflammatory diseases with impact on NF-κB-signaling and apoptosis (73). Here we show that its protein level can be regulated by TTP binding to the 3′UTR of its mRNA, although multiple AREs are also detected within intronic regions of the primary transcript. Interestingly, Tnfaip3/A20 also seems to be post-transcriptionally regulated by Roquin (Rc3h1) through binding to constitutive decay elements (CDEs) within the 3′UTR of its mRNA (74). It is intriguing that two different RBPs can bind to the same mRNAs on neighboring elements. Stringent regulatory mechanisms are needed to orchestrate binding of the two RBPs to different recognition sequences on the same transcript and simultaneous binding can have synergistic or cross-inhibitory effects.

We observed a prolonged phosphorylation of p38^MAPK^ and MK2 suggesting a potential impact of GFP–TTP-AA expression on MAPK-signaling. We presume that TTP could act as a global negative regulator of cell signaling upon LPS stimulation. This hypothesis was supported by gene ontology, gene set enrichment and pathway enrichment analyses that revealed ‘regulation of signal transduction’ processes and ‘cell death’ as common major functions affected by TTP expression. Importantly, changes in these processes can be linked to alterations in NF-κB-signaling that were proven at multiple levels. Altered phosphorylation of NF-κB because of delayed activation of its upstream activators and associated pathways resulted in a decelerated translocation of NF-κB into the nucleus. This block of translocation was accompanied by increased TTP-AA level detected in the nucleus (Supplementary Figure S6b, anti-GFP blots). This supports the observation made in p38^MAPK^-inhibited LPS-stimulated macrophages, where TTP also accumulated in the nucleus (75). A block of NF-κB nuclear translocation mediated by TTP was also suggested previously (24,25). This effect can be explained by post-transcriptional regulation of NF-κB-modulators such as Nfkbia or Nfkbiz and deregulation of feedback inhibitors of NF-κB-activation and MAPK-signaling with known effects on the inflammatory response. However, the phenotype of TTP^aa/aa^ knock-in mice suggested no differences in NF-κB activation, as assessed using reporter assays and by analyzing NF-κB-dependent transcription of TNF (26). Differences to the results presented here may have arisen for several reasons: (i) In our study GFP–TTP-AA is expressed to similar levels as wild-type TTP and, therefore could have exerted more potent effects on feedback regulators than the very low autoregulated level of TTP-AA in the TTP^aa/aa^ mouse (26). Since our TTP-rescue constructs do not contain TTP's 3′UTR, we avoided its reported autoregulation (37) leading to reduced mRNA and protein level in mice (26). Furthermore, the N-terminal GFP-tag of TTP-AA could have alleviated the proteasomal TTP-AA degradation (26,75) that occurs in an ubiquitin-independent manner (76). Therefore, we were able to monitor differences that are only due to phosphorylation and are independent of autoregulation and altered protein stability. (ii) In the TTP^aa/aa^ mouse TNF was investigated as NF-κB responsive gene while our study monitored upstream post-transcriptional events of NF-kB activation.

Deregulation of feedback inhibitors, NF-κB-regulators, cytokines and chemokines is paralleled by an increased apoptosis of TTP-AA expressing cells before as well as during LPS stimulation. Interestingly, the population of apoptotic cells expresses increased amounts of TTP-AA, while non-apoptotic cells display significantly lower TTP-AA levels. This is consistent with observations made by overexpressing TTP in various cell lines leading to increased apoptosis (77). These results also corroborate the observation that in the TTP^aa/aa^ knock-in mouse model the proteasome-dependent inhibition of TTP-AA expression is necessary for viability of the generated animals (26). It may well be that the TTP-AA expressing non-apoptotic cells described here escape from apoptosis by using a similar mechanism.

Unlike Ross *et al*. (26), we achieved expression of TTP-AA protein to similar levels as wild-type TTP by preventing its autoregulation through deletion of its ARE. The levels were also similar to endogenous amounts detected in LPS-stimulated RAW264.7 cells. However, the basal expression of TTP after Dox-induction before LPS stimulation does not reflect the situation for endogenous TTP and could introduce artificial LPS-independent effects on gene expression. However, our main conclusions were drawn from the results generated after 1h of LPS stimulation and should reflect the physiological situation.

The modulation of TTP protein levels could have therapeutic potential in diseases associated with inflammation. As shown by deleting the AREs in TTP's mRNA the destructive binding of TTP to its own mRNA was avoided and resulted in increased endogenous TTP protein level that were beneficial in various disease models in mice (78). Targeted overexpression of TTP-AA could be a new tool to alter cell growth as it was shown for other phosphorylation site mutants of TTP (79). Nonetheless, the modulation of wild-type TTP expression or introduction of mutant TTP protein should be considered with caution, because of the involvement of deregulated TTP in cancer (80), its emerging role as tumor suppressor (81) and the observed high levels of TTP in different late-stage tumor-associated macrophages (82). Taken together, our mechanistic transcriptome-wide studies complement recent mouse models, functional studies at the molecular level and serve to understand the complex landscape of TTP function.

## DATA AVAILABILITY/ACCESSION CODES

Sequencing data is deposited at the Gene Expression Omnibus (GEO) under the identification number GSE81250 and will be deposited for visualization in the TTP atlas (http://ttp-atlas.univie.ac.at) (83).

## Supplementary Material

SUPPLEMENTARY DATA
