# Supplementary material for: The RNA-binding protein TTP is a global post-transcriptional regulator of feedback control in inflammation
Source: Nucleic Acids Res. 2016 May 24;44(15):7418–40. doi: 10.1093/nar/gkw474 (PMC5009735; doi:10.1093/nar/gkw474)
Supplement: SUPPLEMENTARY DATA [file supp_44_15_7418__index.html]

The RNA-binding protein TTP is a global post-transcriptional regulator of feedback control in inflammation — SUPPLEMENTARY DATA 

# The RNA-binding protein TTP is a global post-transcriptional regulator of feedback control in inflammation

## SUPPLEMENTARY DATA

- SUPPLEMENTARY DATA
- SUPPLEMENTARY DATA
- SUPPLEMENTARY DATA
- SUPPLEMENTARY DATA
- SUPPLEMENTARY DATA
- SUPPLEMENTARY DATA
- SUPPLEMENTARY DATA
- SUPPLEMENTARY DATA
