# Supplementary material for: The RNA-binding protein TTP is a global post-transcriptional regulator of feedback control in inflammation
Source: Nucleic Acids Res. 2016 May 24;44(15):7418–40. doi: 10.1093/nar/gkw474 (PMC5009735; doi:10.1093/nar/gkw474)

Supplementary Information

The RNA-binding protein TTP is a global post-transcriptional regulator of feedback control in inflammation

Christopher Tiedje[1,4,*], Manuel D. Diaz-Muñoz[2,4], Philipp Trulley[1], Helena Ahlfors[2], Kathrin Laaß[1], Perry J. Blackshear[3], Martin Turner[2] and Matthias Gaestel[1]

[1]Institute of Physiological Chemistry, Medical School Hannover (MHH), 30625 Hannover, Germany

[2]Lymphocyte Signalling and Development, The Babraham Institute, CB22 3AT, Cambridge, UK

[3]Laboratory of Signal Transduction, National Institute of Environmental Health Sciences, Research Triangle Park, North Carolina 27709, USA; and Departments of Medicine and Biochemistry, Duke University Medical Center, Durham, North Carolina 27710, USA

[4] co-first authors

*Correspondence: Tiedje.Christopher@mh-hannover.de

Running title: TTP controls feedback regulators

Key words: iCLIP, TTP, MAPKAPK2, phosphorylation, NF-κB

Contains:

Supplementary Figure Legends

Supplementary Tables and Legends

Supplementary source data sets descriptions

Supplementary Figure 1

Comparison of expression of TTP in transduced macrophages and RAW264.7 cells

(a) Detection of LPS-induced expression of TTP in RAW264.7 cells compared to the immortalized TTP-deficient BMDM cell line using two different TTP-specific antibodies (SAK21B, pan-Tis11). Various signals obtained with the anti-pan-Tis11 antibody probably belong to the other Zfp36 family member proteins Zfp36l1 and Zfp36l2. The arrow indicates the position of endogenous TTP.

(b) Macrophages expressing GFP-TTP and GFP-TTP-AA upon doxycycline addition for 6h were positively sorted using FACS. Representative FACS plots of transduced non-sorted immortalized TTP-deficient BMDMs stimulated or not with 1µg/ml doxycycline for 6h.

(c) Quantification of GFP mean fluorescence intensity (MFI) of the FACS experiments are shown in the bar graph diagram and demonstrate that both GFP-TTP and GFP-TTP-AA were expressed to a similar extent.

(d-h). GFP-TTP and GFP-TTP-AA were expressed with similar kinetics and similar effects on TNF expression.

(d) Quantification of GFP-TTP and GFP-TTP-AA protein levels as observed in Figure 1c, where expression of both proteins was induced with doxycycline for different times and combined with LPS stimulation. TTP signals were quantified and normalized to GAPDH signals. Relative normalized intensities are shown with standard deviations.

(e) Comparison of TTP expression in LPS-treated RAW264.7 cell and in Doxycycline/LPS-treated GFP-TTP expressing macrophages by LiCOR Western blot analysis. Western blot (upper panel) and quantification of signals (lower panel) are shown. Signals related to TTP obtained from different exposures were normalized to β-Actin. Ratios are plotted against LPS (for RAW264.7 cells) or combined LPS and doxycycline (for GFP-TTP expressing cells) stimulation time. This kinetic analyses of GFP-TTP expression in macrophages revealed that a combined treatment with doxycycline for 4 to 6h and LPS for the last 1h mimicked the expression and phosphorylation of TTP in RAW264.7 monocytes after 90 to 120 min treatment with LPS.

(f) TNF mRNA levels were determined by qPCR in the cell lines described in Figure 1a-c. 6h doxycycline induction was combined with the indicated times of LPS stimulation. The rescue of TTP activity in the inducible cell system was achieved as macrophages expressing either GFP-TTP or GFP-TTP-AA had a profound reduction in TNF mRNA after LPS stimulation compared to TTP-deficient macrophages expressing only GFP (GFP/EV). The impact of GFP-TTP-AA expression on TNF mRNA was markedly more pronounced than for GFP-TTP expression. The asterisks (*) indicate $p \leq 0.05$.

(g) TNF mRNA stability was monitored by qPCR for the indicated conditions and could explain changes in TNF mRNA level (Fig. S1f) by changes in mRNA stabilities. RNA Polymerase II-mediated transcription was blocked by treating the cells with 10µg/ml Actinomycin D (ActD) after initial stimulation. Samples were analyzed every 15 minutes. Calculated mRNA half-lives are shown in the legend.

(h) Western blot analyses showing the induction of pro-TNF protein biosynthesis by LPS and its reduction in dependence of GFP-TTP or GFP-TTP-AA expression connecting changes in mRNA level and changes in mRNA stabilities to changes in TNF protein synthesis. GAPDH served as loading control. Quantifications of Western blot signals normalized to GAPDH signals are shown in the lower panel. The asterisks (*) indicate $p \leq 0.05$.

Supplementary Figure 2

iCLIP library construction

(a) GFP and GFP-TTP were precipitated (IP) under native (N) and 2M urea (U) conditions by GFP-Nanotrap pulldown and analyzed by ECL-based Western blot. The input for each IP is shown left of the IP lanes.
(b) GFP-TTP precipitates were treated with different RNases for 5 min at 37°C. After labelling the RNAs with radioactive ^{33}P, a 4-12% NuPAGE gradient gel was used to resolve GFP-TTP-RNA complexes that were transferred to a nitrocellulose membrane afterwards. The autoradiograph is shown. RNase A was the only RNase that digested TTP-associated RNAs to a minimal size under denaturing conditions.
(c) Precipitated radiolabelled GFP-TTP-RNA and GFP-TTP-AA-RNA complexes after resolution by gel electrophoresis. Several concentrations of RNase A were tested in order to isolated TTP-RNA complexes above 100 kDa for iCLIP library preparation ("cut"). Importantly, immunoprecipitation using protein lysates from GFP-only expressing control macrophages did not result in co-purification of RNAs for cDNA library preparation (data not shown).

Supplementary Figure 3

Peak and density enrichment analysis of the genomic distribution of iCLIP crosslinks in dependence on the stimulation

(a-c) The detailed distributions of iCLIP targets (peak enrichment) from GFP-TTP and GFP-TTP-AA expressing cells stimulated as indicated above the plots are shown as mean and standard deviation. For 6h/1h LPS (a) four (GFP-TTP) and three (GFP-TTP-AA) replicates were considered, respectively. For all other conditions (6h Dox (b) and 6h Dox/4h LPS (c)) the mean and standard deviations of data from two individual experiments are shown.
(d,e) Complementary to the density enrichment for 6h Dox and 1h LPS shown in Figure 1d, density enrichments for the genomic features that are crosslinked to GFP-TTP and GFP-TTP-AA are depicted for 6h Dox (d) and 6h Dox/4h LPS (e).

Supplementary Figure 4

Confirmation of TTP and TTP-AA binding to novel targets

(a) Examples of precipitates of GFP, GFP-TTP and GFP-TTP-AA used for RNA-IP experiments (Figs. 3a-c and 5a-c).
(b) Insertion of 3´UTRs of Cxcl10 and Gdf15 mRNAs reduce the expression of Luciferase reporters in HeLa cells. * $p \leq 0.05$.
(c) Luciferase reporter expression using the 3´UTRs of Cxcl10 and Gdf15 was further decreased by co-transfection of either GFP-TTP or GFP-TTP-AA. A significantly enhanced reduction by GFP-TTP-AA compared to GFP-TTP was only observed for Gdf15. * $p \leq 0.05$.
(d) Reporter gene assay as for Cxcl10 and Gdf15 in (b) was done for the insertion of the Tnfaip3 3´UTR. * $p \leq 0.05$.
(e) Reporter gene assays as in (b) and (c) for the 3'UTR of Tnfaip3 mRNA. * $p \leq 0.05$.

Supplementary Figure 5

Analysis of the translational regulation of TTP targets using ribosome profiling

Fractionation of ribosome-associated mRNAs was performed using sucrose gradient centrifugation followed by gradient fractionation and quantification of specific mRNAs by RT-qPCR in the different fractions. The repression of polysomal loading differed from transcript to transcript.
(a) Examples of polysome gradients for the different cells and the indicated conditions. Absorption at 254 nm is plotted against the fraction number.

(b) Association of TNF mRNA (left panels) with actively translating ribosomes (fractions 6-11) is shown by polysome profiling under the indicated conditions. For each fraction percentages in relation to relative total mRNA amounts of all fractions for each transcript were calculated and plotted against the fraction numbers. Expression of TTP or TTP-AA resulted in a clear reduction of polysomal loading of TNF mRNA and a shift to monosomal fractions (2-5). The corresponding distribution of β-Actin control mRNA is shown on the right; it is not affected by TTP or TTP-AA expression.

(c) The distribution of inflammation-related mRNAs for Cxcl10 (left) and Gdf15 (right) were analyzed in polysome profiling experiments. Reduction of polysomal loading by TTP or TTP-AA expression was evident for Gdf15.

(d) Distribution of mRNAs for the following feedback inhibitors was analyzed by polysome profiling experiments under the indicated conditions: Dusp1 (left), Ier3 (middle) and Tnfaip3 (right panels) mRNAs. The most prominent effects by TTP or TTP-AA expression were observed for Ier3 mRNA after 1 or 2h LPS treatment.

Supplementary Figure 6

NF-κB-related proteins in dependence of TTP- and TTP-AA-expression

TTP- and TTP-AA-expression influence NF-κB activation and signaling**.** For quantification of this effect Western blot signals derived from Figure 4c and Figure S6 b, a minimum of two different exposures was normalized to at least three different exposures of the loading control blot that was derived from the same membrane. All background-reduced band intensities were determined using the Multi Gauge V3.2 software (FujiFilm).

(a) Quantification of the Western Blot signals derived from Figure 4c, where cells of all three genotypes were pre-treated with doxycycline for 6h and consequently stimulated with LPS for 0, 15, 30, 60, 120 and 240 minutes. GAPDH served as loading control and was used for normalization of specific signals.

(b) The NF-κB distribution upon differential lysis was analyzed by Western Blot. For this, nuclear and cytosolic fractions of the different cell types were analyzed for the indicated proteins in a time-dependent manner. Tubulin served as a cytosolic and ATF-2 as a nuclear marker.

(c) Effect of TTP/TTP-AA expression on the nuclear translocation of NF-κB. The nuclear to cytosolic ratio of NF-κB Western blot signals derived from (b) were calculated and plotted against the time of LPS treatment.

Supplementary Figure 7

RNASeq and RiboSeq analyses reveal changes in the cytokine signatures of GFP-TTP-AA cells

(a-b) Heatmaps for the expression profiles of RNAseq- (a) and RiboSeq- (b) derived data limited to the enriched gene set “cytokine activity” (GO:0005125) that includes chemokines. Each heatmap shows three replicates of GFP/EV-, GFP-TTP- and GFP-TTP-AA-expressing cells as a single row each. Row z-score distributions are shown in the upper right corner of each map. Cluster analysis of RNASeq and RiboSeq data reveals that GFP-TTP-AA expressing cells cluster separately in this analysis, while GFP- or TTP-expressing cells cluster together.

Supplementary Figure 8

Quantification of feedback inhibitor proteins

TTP- and TTP-AA-expression modulate the expression of feedback inhibitors to different extent.

(a-c) Quantification of Western blot signals from Figure 5f. a) shows Dusp1, b) Ier3 and c) Tnfaip3 protein levels. GAPDH served as loading control and was used for normalization of specific signals.

Normalized signals are shown as mean with their standard deviations. * $p \leq 0.05$. For quantification of Western blot signals a minimum of two different exposures was normalized to at least three different exposures of the loading control blot that was derived from the same membrane. All background-reduced band intensities were determined using the Multi Gauge V3.2 software (FujiFilm). The signals of GFP/EV-expressing cells were set to one in unstimulated and 6h Dox/2h LPS stimulated cells.

Supplementary Figure 9

A similar role of TTP and TTP-AA in Ier3- and Dusp1-expression of mouse embryonic fibroblasts (MEFs)

(a) Sorted immortalized TTP-deficient MEFs transduced with the same vectors like the immortalized BMDMs were induced for expression of GFP, GFP-TTP and GFP-TTP-AA with doxycycline for a total of 6h. Cells were starved for 16h in serum-free medium and phosphorylation of TTP was induced by stimulating the cells for 90 min with 10% (v/v) FCS to activate the $p38^{MAPK}$/MK2 signaling pathway. Activated $p38^{MAPK}$ and its downstream kinase MK2 are detected by phospho-specific antibodies as indicated. Phosphorylated TTP was detected by the $pTTP^{S178}$-specific antibody. GAPDH served as loading control.

(b) Changes in Ier3 mRNA levels were measured in the TTP KO MEF cell lines. Cells were starved in serum-free medium overnight before inducing GFP/EV, GFP-TTP or GFP-TTP-AA expression for 4.5h with doxycycline. Then, cells were stimulated with 10% (v/v) FCS for 90 min, before the cells were harvested for mRNA expression analysis by qPCR. Values are shown as mean with their standard deviations. Changes in Dusp1 and Cxcl10 mRNA level were measured from the samples and showed similar TTP-dependence (data not shown).

(c) Changes in Ier3 and Dusp1 protein levels were detected by Western blot under the same conditions as in (b). The arrow indicates for the position of Ier3 protein. Other bands in the same blot were unspecific. GAPDH served as a loading control.

(d) and (e) Western blot signals from (c) were quantified. A minimum of two different exposures was normalized to at least three different exposures of the loading control blot that was derived from the same membrane. All background-reduced band intensities were determined using the Multi Gauge V3.2 software (FujiFilm). Protein levels for each genotype under unstimulated conditions were set to one. Quantified signals of Ier3 protein level (e) and Dusp1 (f) are shown as mean and standard deviations. * $p \leq 0.05$. Quantification

Supplementary Table 1

Distribution of cDNA counts, number of overlapping targets and percentages of them comparing GFP-TTP and GFP-TTP-AA 3´UTR iCLIP targets upon 6h doxycycline induction in combination with 1h LPS stimulation. Targets with >1, ≥5 or ≥10 cDNA counts in their 3´UTR were compared.

6h Dox/1h LPS	> 1 count	≥ 5 counts	≥ 10 counts
TTP iCLIP 3´UTR targets	1112	290	135
TTP-AA iCLIP 3´UTR targets	3763	2323	1581
overlapping targets	953	279	131
overlapping targets [%]	85.7	96.21	97.04
individual TTP iCLIP 3´UTR targets	159	11	4
individual TTP-AA iCLIP 3´UTR targets	2810	2044	1450

Supplementary Table 2

Summary of the high-throughput sequencing approaches used in this study. For each method the samples that were analyzed and the different conditions for each sample are listed.

Method	Samples	Condition: 6h Dox +	Replicates
**iCLIP**	GFP-TTP, GFP-TTP-AA	1h LPS, 4h LPS, no LPS	4/3, 2/2, 2/2
**RNASeq**	GFP/EV, GFP-TTP, GFP-TTP-AA	1h LPS	3/3/3
**RiboSeq**	GFP/EV, GFP-TTP, GFP-TTP-AA	1h LPS	3/3/3

Supplementary Table 3

Enriched pathways for differentially expressed (DE) genes determined by RNASeq for cells expressing GFP/EV (GFP), GFP-TTP (TTP) or GFP-TTP-AA (AA) upon 6hDox/1h LPS using WebGestalt. The number of reference genes associated to each pathway, the number of DE genes enriched in each pathway, as well as the adjusted p-value (hypergeometric test and Benjamini-Hochberg multiple test correction) for each enriched pathway are shown. PluriNetWork, mechanisms associated with pluripotency; MAPK, mitogen-activated protein kinase; EGFR1, epidermal growth factor receptor 1. NF-κB, nuclear factor "kappa-light-chain-enhancer" of activated B-cells. Enriched pathways related to signaling are highlighted in bold.

**1. RNASeq TTP vs. GFP**			
**Pathway Name**	**# reference genes**	**# DE genes**	**adjusted p-value**
mRNA processing	483	34	1.33E-9
PluriNetWork	292	22	5.92E-7
G1 to S cell cycle control	65	10	3.95E-6
**Chemokine signaling pathway**	186	15	2.01E-5
**IL-3 Signaling Pathway**	111	11	5.33E-5
DNA Replication	41	7	5.59E-5
Focal Adhesion	186	14	5.59E-5
**EGFR1 Signaling Pathway**	217	15	5.87E-5
**TNF-alpha NF-kB Signaling Pathway**	215	15	5.87E-5
Calcium Regulation in the Cardiac Cell	152	12	0.0001
**2. RNASeq TTP-AA vs. GFP**			
**Pathway Name**	**# reference genes**	**# DE genes**	**adjusted p-value**
**TNF-alpha NF-kB Signaling Pathway**	215	37	1.14E-12
**Insulin Signaling**	158	30	1.25E-11
mRNA processing	483	55	1.49E-11
Focal Adhesion	186	32	1.63E-11
**MAPK signaling pathway**	165	30	1.63E-11
PluriNetWork	292	40	3.58E-11
**B Cell Receptor Signaling Pathway**	202	32	9.79E-11
**IL-3 Signaling Pathway**	111	22	1.92E-9
**EGFR1 Signaling Pathway**	217	31	2.36E-9
Myometrial Relaxation and Contraction Pathways	158	26	2.48E-9
**3. RNASeq TTP-AA vs. TTP**			
**Pathway Name**	**# reference genes**	**# DE genes**	**adjusted p-value**
mRNA processing	483	128	5.95E-45
PluriNetWork	292	73	6.24E-24
**TNF-alpha NF-kB Signaling Pathway**	215	58	6.68E-21
**B Cell Receptor Signaling Pathway**	202	54	2.22E-19
**T Cell Receptor Signaling Pathway**	143	45	2.42E-19
**IL-3 Signaling Pathway**	111	37	6.03E-17
Focal Adhesion	186	48	8.61E-17

Supplementary Table 4

Sequences of primers used for genotyping, cloning and qPCR.

Genotyping	Sequence 5´-3´
TTPd	GAG GGC CGA AGC TGC GGC TGG GT
TTPrc1	GGC TGG CCA GGG AGA GCT AGG TC
Tneorc	CTG TTG TGC CCA GTC ATA GCC G
**Cloning**	**Sequence 5´-3´**
TTP-22-fwd	CATGTGTACACTAATCCGGATTAATTAATTCATTA
TTP-23-rev	CATGTGTACAAGATGGATCTCTCTGCCATCTA
Tnfaip3-1-fwd	GGACTAGTGTGCGAACACATTGACAGG
Tnfaip3-rev	GGACTAGTTTTTTTTTTTTTTTTTTTGTTCTTAAATAATTTATTTTTTAATATTGAC
Cxcl10-1-fwd	GCTCTAGACTGGAGTGAAGCCACG
Cxcl10-2-rev	GCTCTAGATTTTTTTTTTTTTTTTTTTTTTCATGTCAGTTATTATT
Gdf15-1-fwd	GCTCTAGAGCACCGGGCCCTGCTC
Gdf15-2-rev	GCTCTAGAAGAGGCCAAGCTCACCTTTATTAGCAGAG
**Taq Man - qPCR**	**Sequence 5´-3´**
beta-Actin	4352341E Applied Biosystems, VIC-labelled
TNF-fwd	CAT CTT CTC AAA ATT CGA GTG ACA A
TNF-rev	TGG GAG TAG ACA AGG TAC AAC CC
TNF-probe	FAM – CAC GTC GTA GCA AAC CAC CAA GTG GA – BHQ1
**SYBR Green - qPCR**	**Sequence 5´-3´**
Cxcl10-fwd	TCAGCACCATGAACCCAAG
Cxcl10-rev	CTATGGCCCTCATTCTCACTG
Ier3-fwd	AAGGGTGCTCTACCCTCGAGT
Ier3-rev	GCAGAAATGGGCTCAGGTGT
Dusp1-fwd	TTCTCCAAGGAGGATATGAAGCGT
Dusp1-rev	CTGCATCCGGATTCTGCACT
TNF-fwd	TGCCTATGTCTCAGCCTCTTC
TNF-rev	GAGGCCATTTGGGAACTTCT
Tnfaip3-fwd	CAGAGCAGGGACAAGCAAGTG
Tnfaip3-rev	CTTTCGCAGAGGCAGTAACAGA
Gdf15-fwd	GGCTGCATGCCAACCA
Gdf15-rev	TCTCACCTCTGGACTGAGTATTCC

Supplementary Source Data

Supplementary Data Set 1

Data Set 1 shows all crosslinked target RNAs of the GFP-TTP/GFP-TTP-AA group upon 6h doxycycline stimulation in combination with 1h LPS stimulation sorted by genomic feature.

Supplementary Data Set 2

Tab-formatted RNASeq DESeq2 results.

Supplementary Data Set 3

Tab-formatted RiboSeq DESeq2 results.

Supplementary Data Set 4

DE genes from RNASeq and RiboSeq analyses are sorted whether they are regulated at the level of stability (DE RNASeq only), at the level of translation (DE RiboSeq only) or at both levels (DE RNASeq and RiboSeq). Additionally the iCLIP targets within each group are listed.

Supplementary Data Set 5

Composition of the compiled TNF-/NF-κB custom gene set and the associated RNASeq and RiboSeq DESeq2 analyses.

Supplementary Files 1 and 2

These two bed files summarize iCLIP mapped data for the groups of GFP-TTP (1) and GFP-TTP-AA (2) upon 6h Dox/1h LPS after peak enrichment (FDR<0.05) and allow the upload of the tracks in the UCSD Genome Browser to visualize crosslink events for both proteins.

Supplementary Figure 1

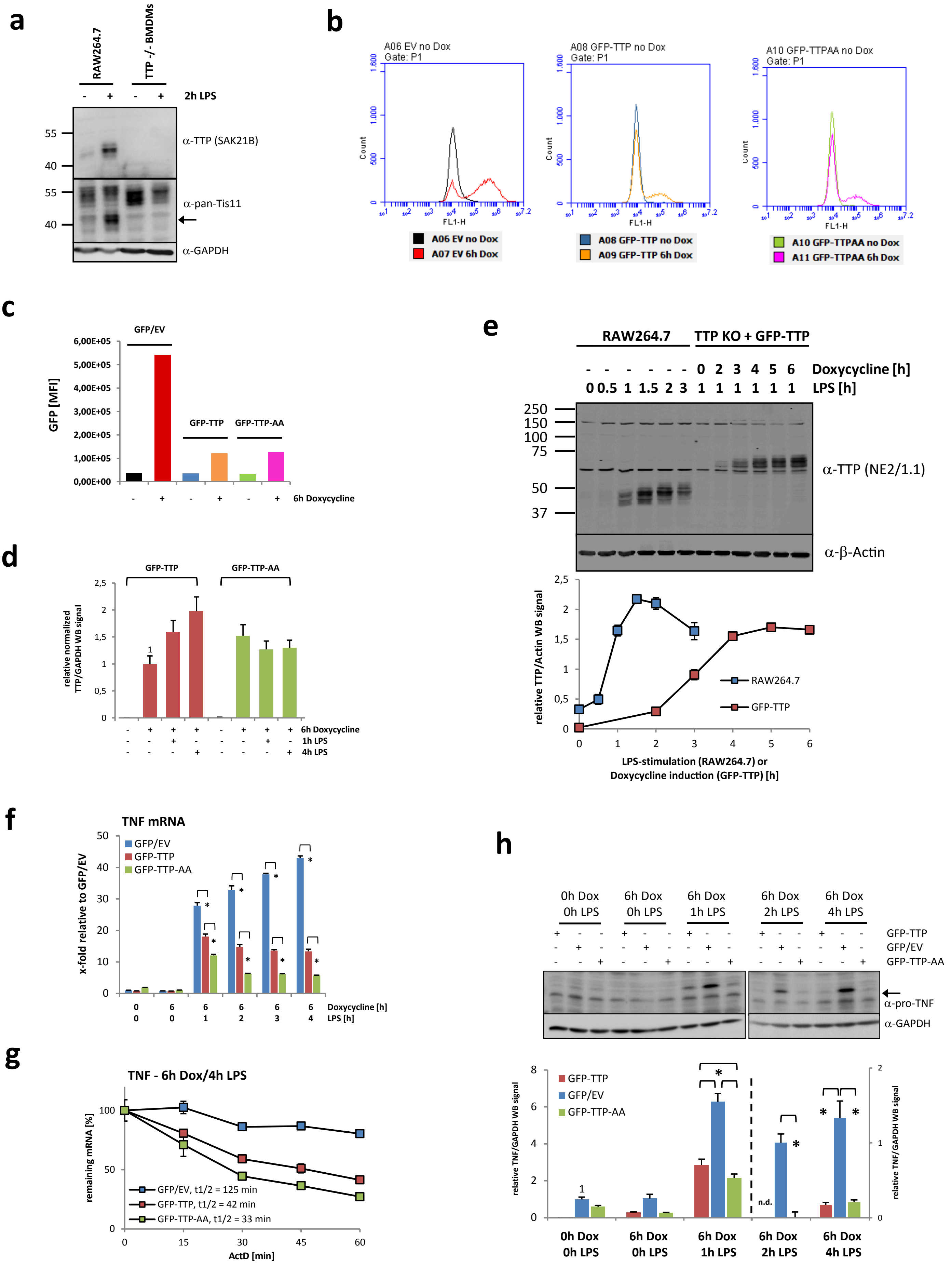

Supplementary Figure 2

a

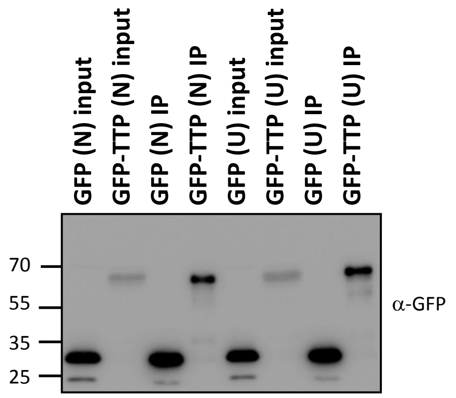

b

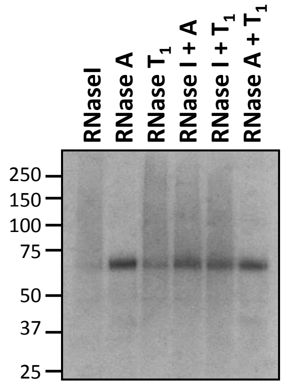

c

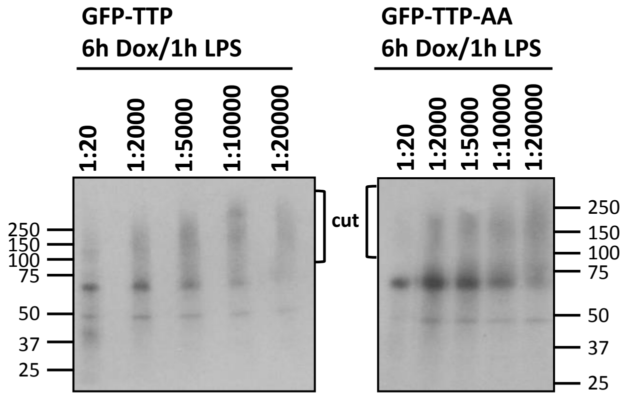

Supplementary Figure 3

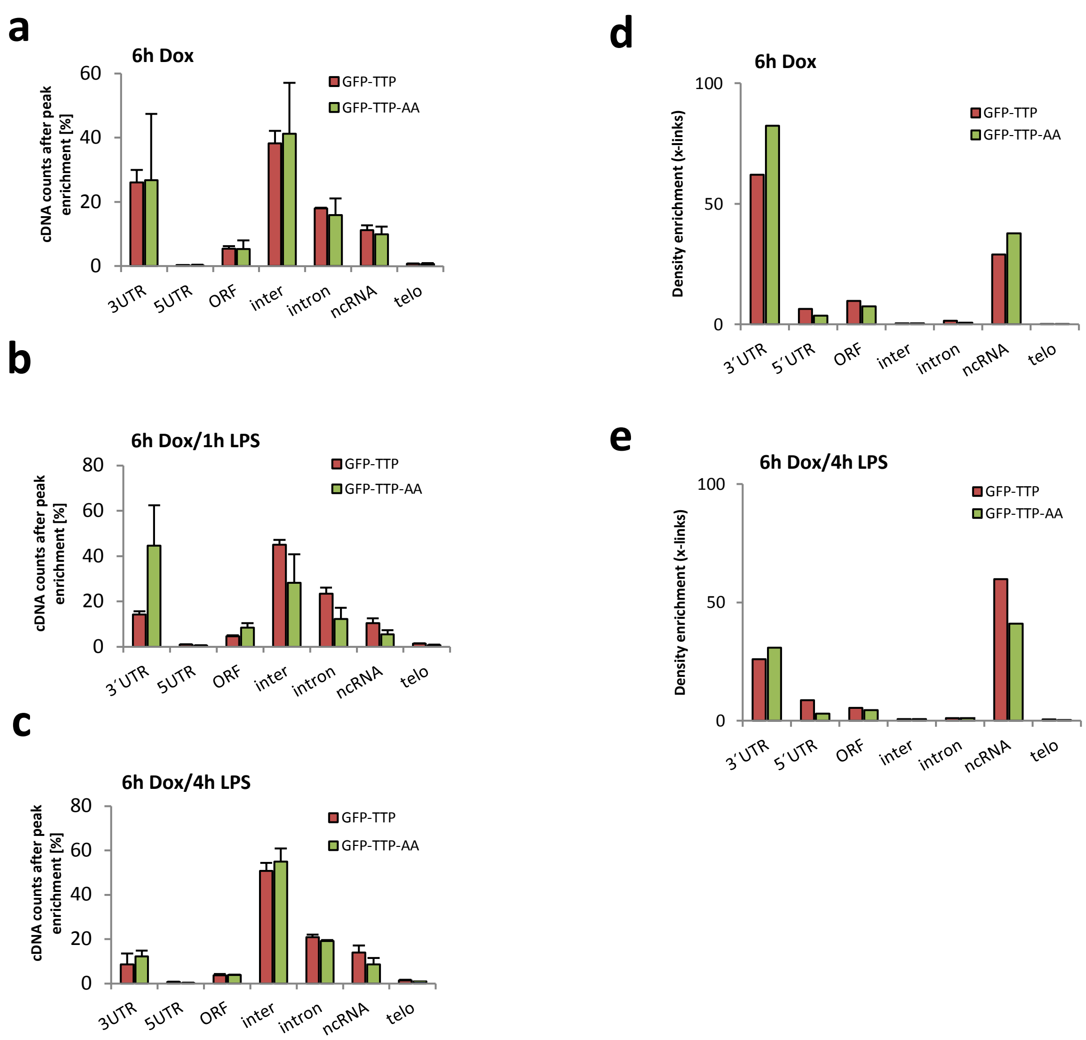

Supplementary Figure 4

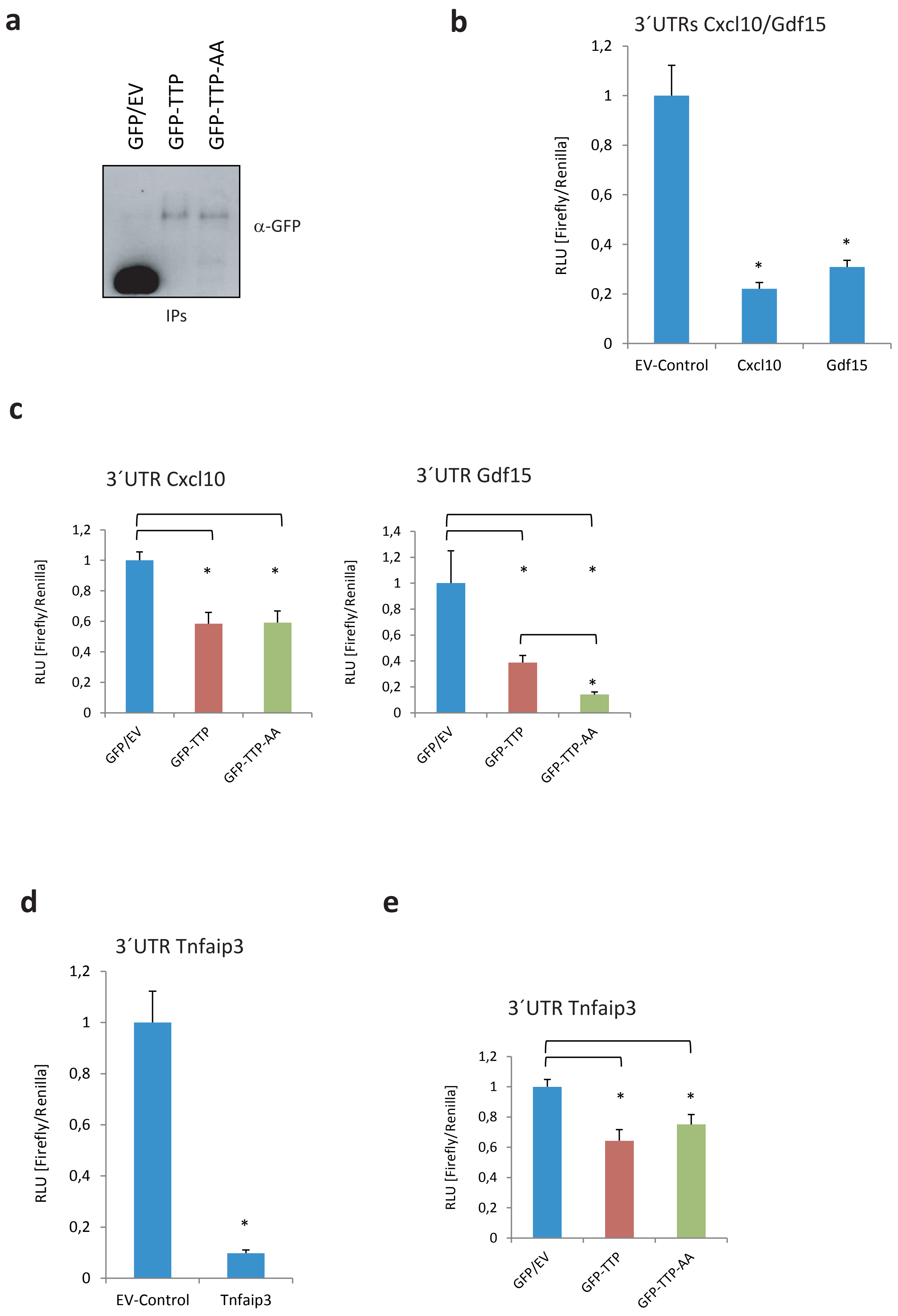

Supplementary Figure 5

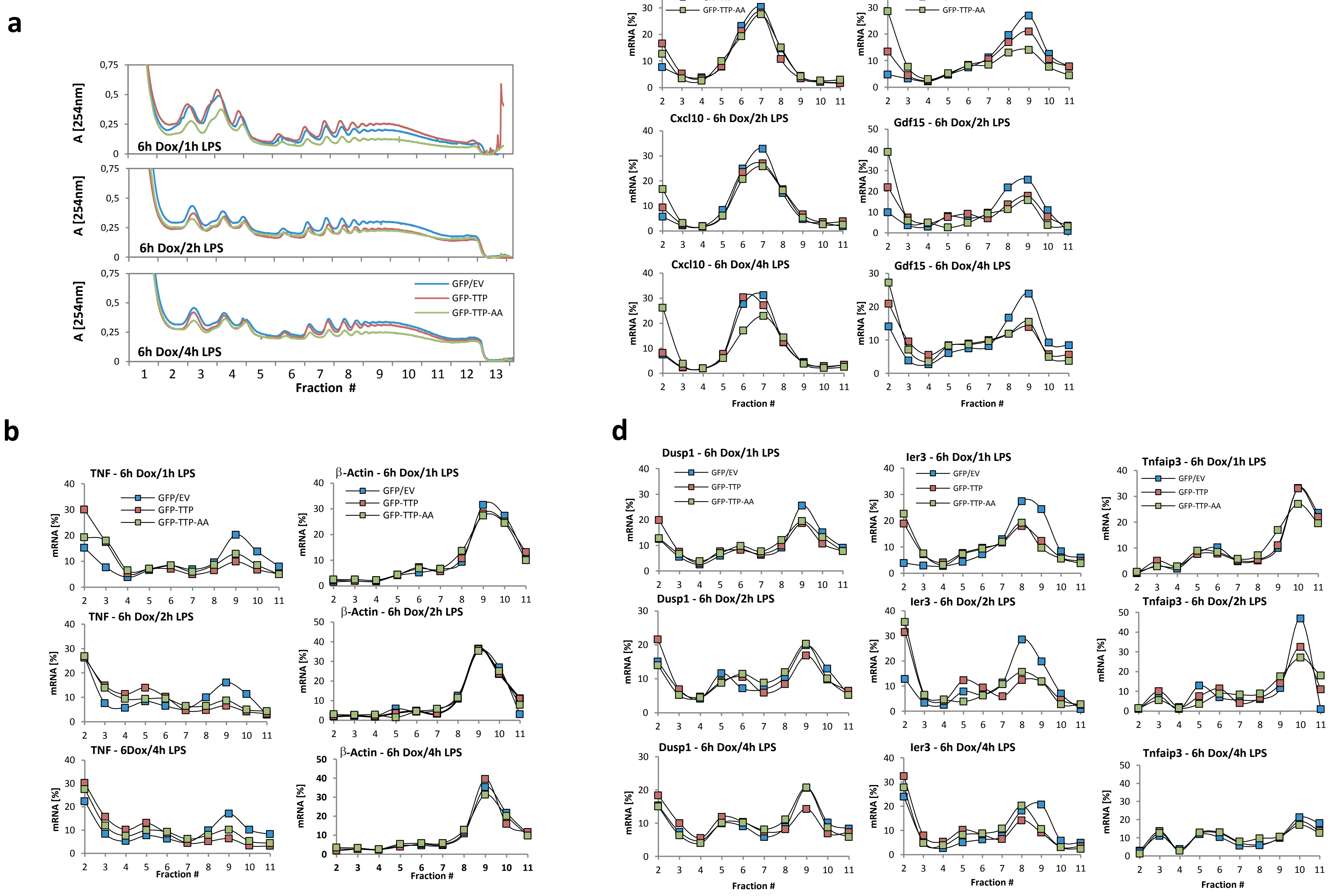

Supplementary Figure 6

a

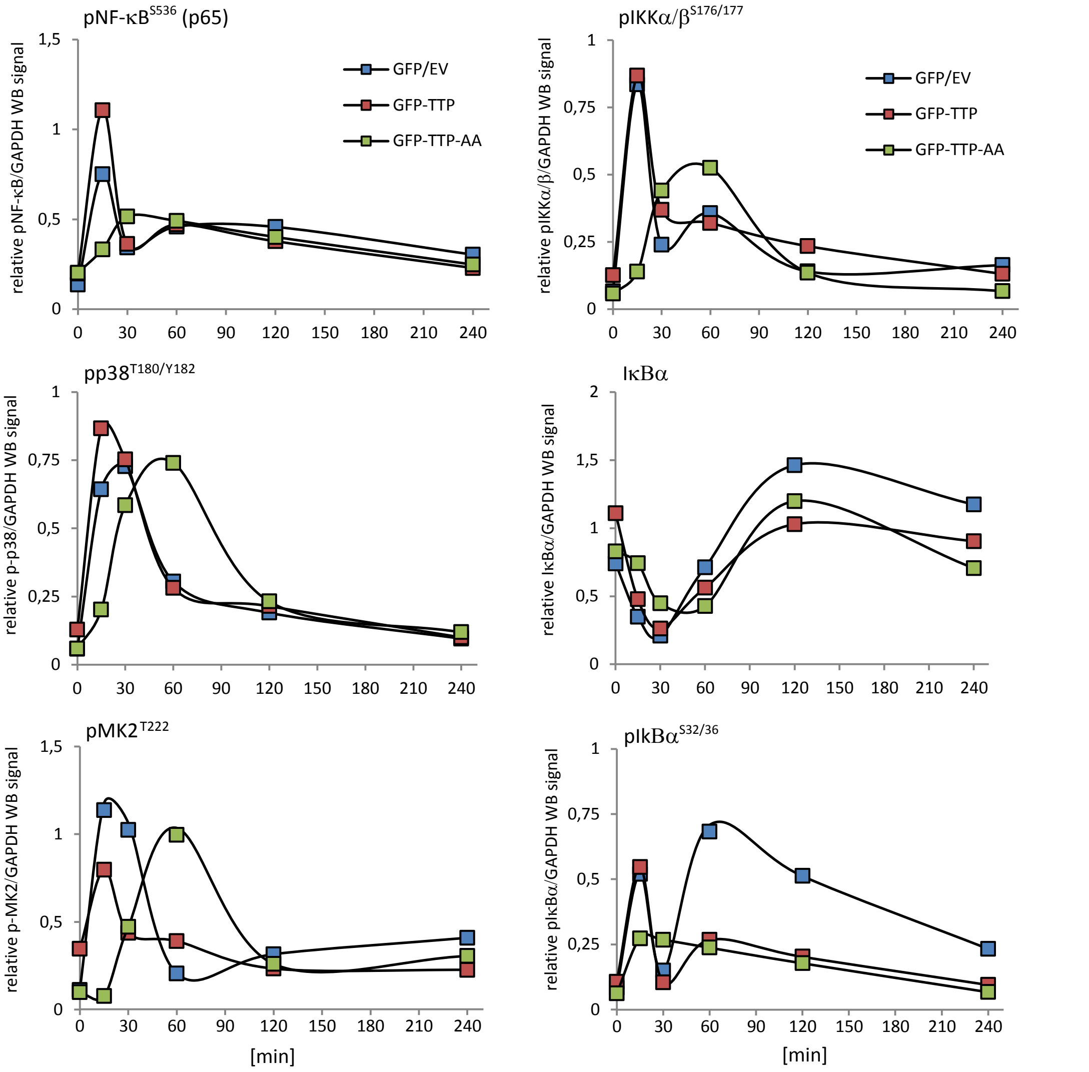

b

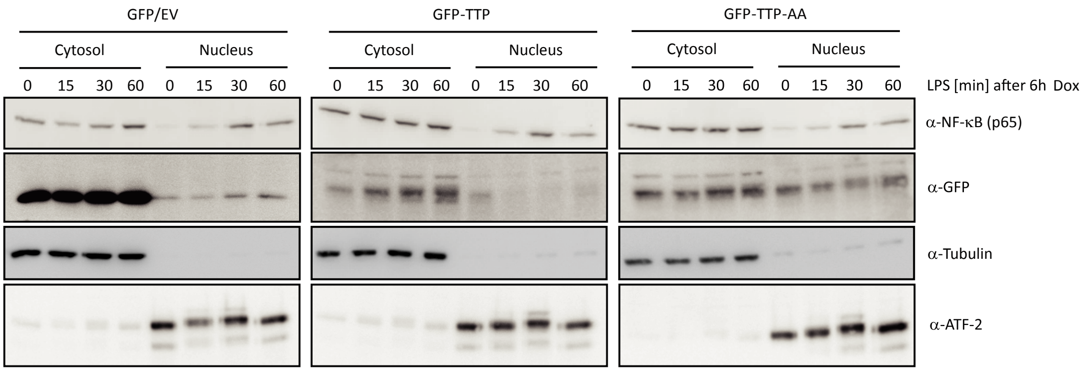

c

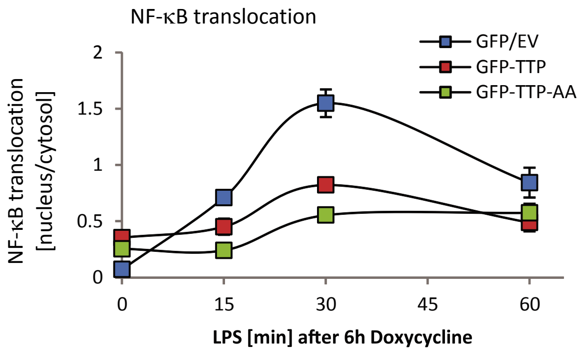

Supplementary Figure 7

a

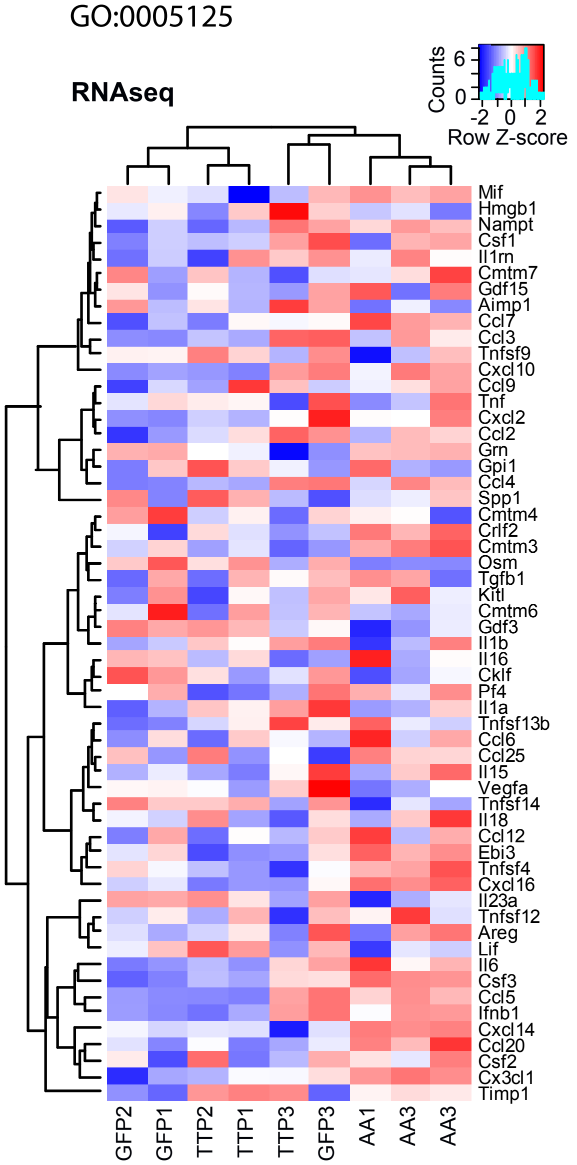

b

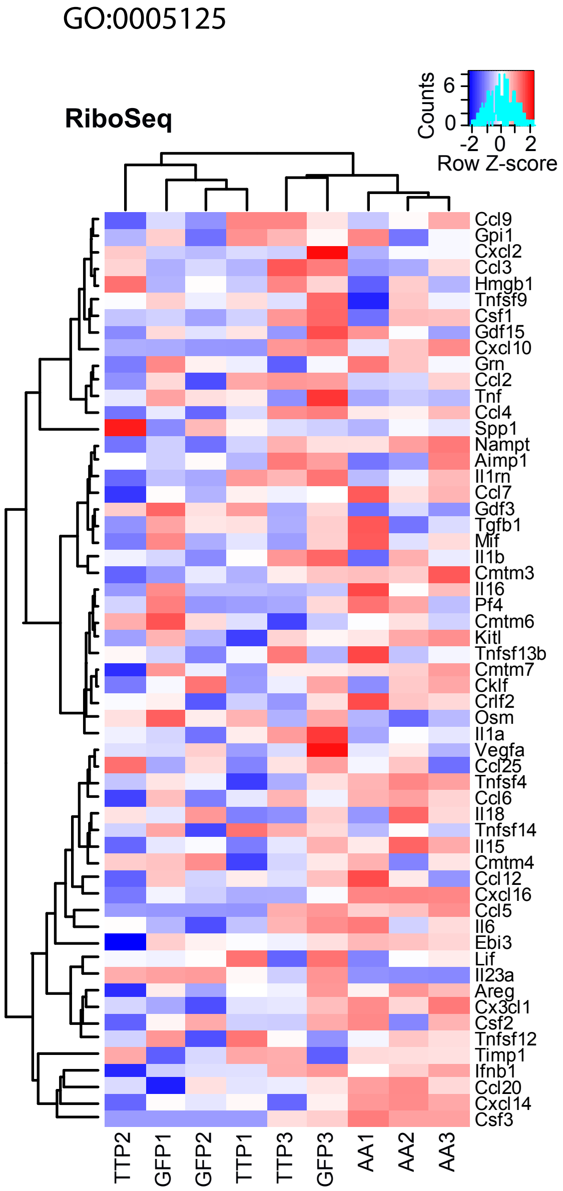

Supplementary Figure 8

a

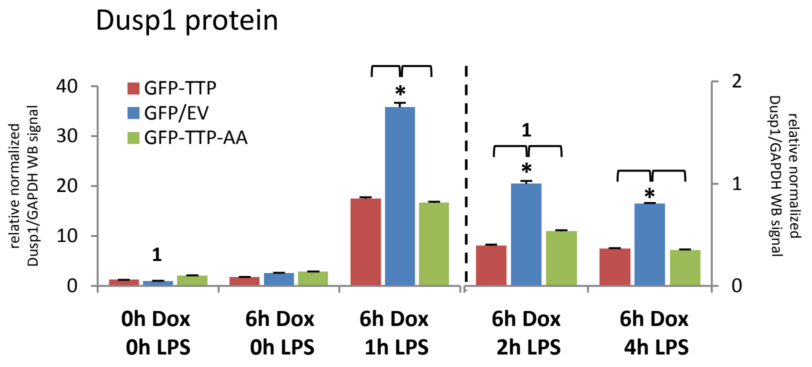

b

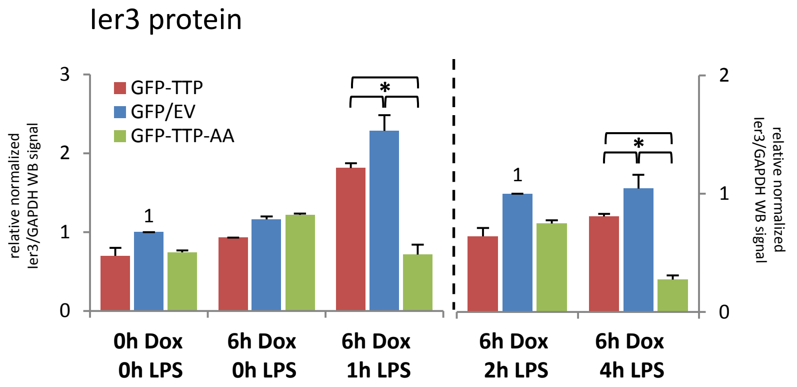

c

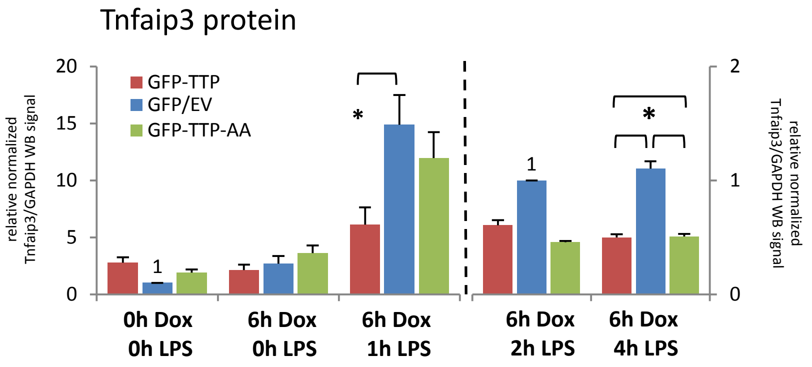

Supplementary Figure 9

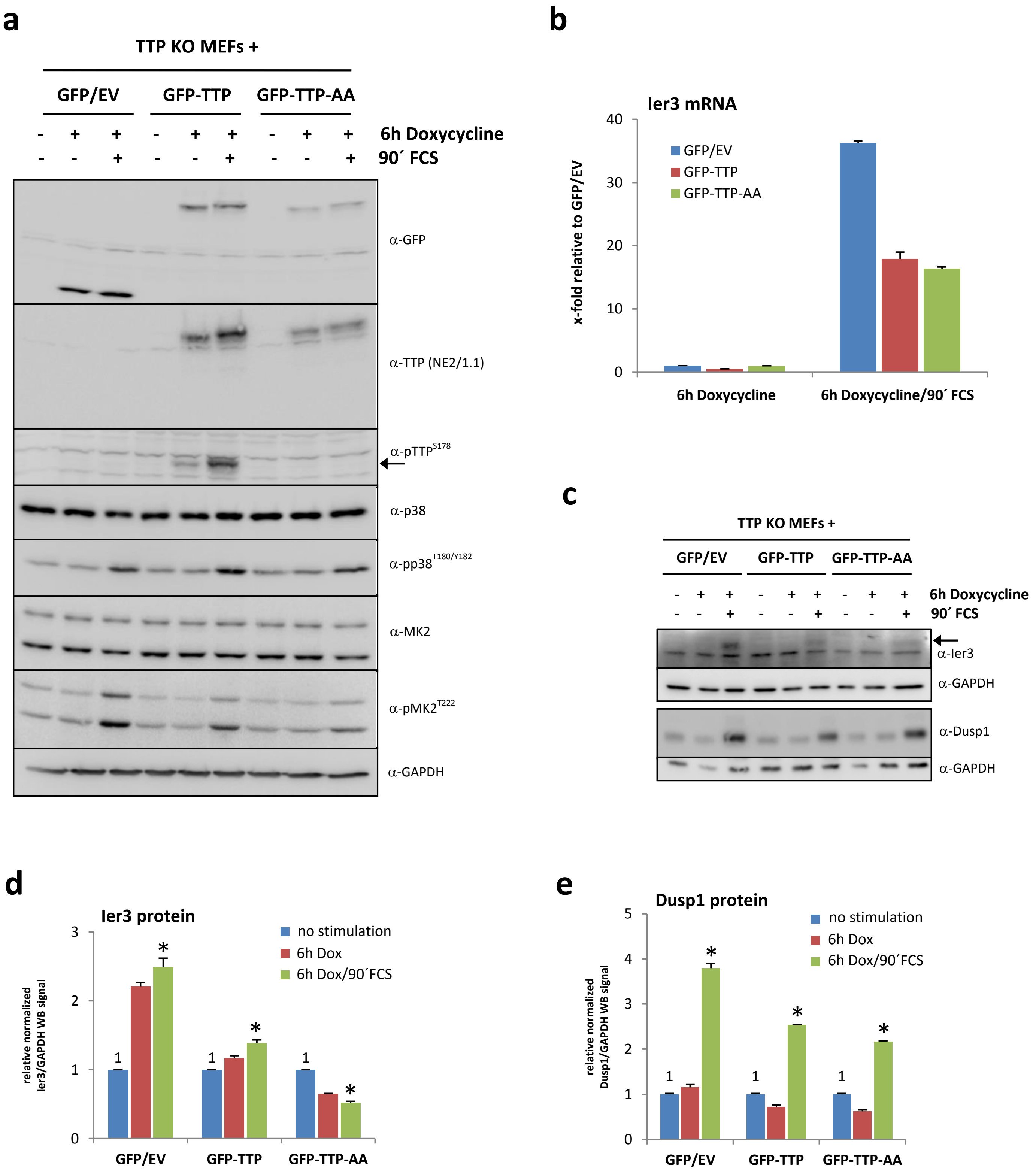

Supplement: SUPPLEMENTARY DATA [file supp_gkw474_nar-00263-y-2016-File010.pdf]
